# Clinical Features and Dental Pathologies in Maxillary Sinus Fungal Balls and Odontogenic Sinusitis

**DOI:** 10.1002/lary.70429

**Published:** 2026-02-07

**Authors:** Eunice Im, Lane Donaldson, Avraham Adelman, Nithin D. Adappa, Yi‐Wei Chen, Nikita Chapurin, Jennifer E. Douglas, Glen E. D'Souza, Jacob Eide, Maria Espinosa, Chia‐Hsiang Fu, Meha G. Fox, Rohit Garg, Edward C. Kuan, Michael A. Kohanski, Marta Kwiatkowska, Kevin Li, Derek H. Liu, Tran Bao Locke, Chih‐Feng Lin, Chadi Makary, Alice Ottavi, Peter Papagiannopoulos, James N. Palmer, Charles C. L. Tong, Bobby A. Tajudeen, Sanjena Venkatesh, Kimberly Wei, Frederick Yoo, Alison J. Yu, Jun Jin, Alberto M. Saibene, John R. Craig

**Affiliations:** ^1^ Michigan State University College of Human Medicine Grand Rapids Michigan USA; ^2^ Department of Otolaryngology—Head and Neck Surgery Henry Ford Health Detroit Michigan USA; ^3^ Department of Otolaryngology—Head and Neck Surgery University of Florida Gainesville Florida USA; ^4^ Division of Rhinology and Skull Base Surgery, Department of Otorhinolaryngology—Head & Neck Surgery University of Pennsylvania Philadelphia Pennsylvania USA; ^5^ Division of Rhinology Linkou Chang Gung Memorial Hospital Taoyuan Taiwan; ^6^ Monell Chemical Senses Center Philadelphia Pennsylvania USA; ^7^ Department of Otolaryngology—Head and Neck Surgery Rush University Medical Center Chicago Illinois USA; ^8^ Department of Otolaryngology—Head and Neck Surgery Mayo Clinic Rochester Minnesota USA; ^9^ Graduate Institute of Clinical Medical Sciences, College of Medicine Chang Gung University Taoyuan Taiwan; ^10^ Department of Otolaryngology—Head and Neck Surgery Baylor College of Medicine Houston Texas USA; ^11^ Department of Otolaryngology—Head and Neck Surgery Kaiser Permanente Orange County Anaheim California USA; ^12^ Department of Otolaryngology—Head and Neck Surgery University of California Irvine Orange California USA; ^13^ Department of Otolaryngology and Laryngological Oncology Military Institute of Medicine—National Research Institute Warsaw Poland; ^14^ Department of Otolaryngology—Head and Neck Surgery National Taiwan University Hospital Taipei Taiwan; ^15^ Department of Otolaryngology—Head and Neck Surgery West Virginia University Morgantown West Virginia USA; ^16^ Department of Health Sciences, San Paolo and Carlo Hospital, Otolaryngology Unit University of Milan Milan Italy; ^17^ Department of Otolaryngology Donald and Barbara Zucker School of Medicine at Hofstra/Northwell New York New York USA; ^18^ Department of Public Health Sciences Henry Ford Health System Detroit Michigan USA; ^19^ Department of Epidemiology and Biostatistics Michigan State University East Lansing Michigan USA; ^20^ Department of Otolaryngology Michigan State University College of Human Medicine Lansing Michigan USA

**Keywords:** apical periodontitis, dental implant, endoscopic sinus surgery, fungal ball, odontogenic sinusitis, oroantral fistula, root canal treatment

## Abstract

**Objectives:**

While maxillary sinus fungal balls (MSFB) can be associated with odontogenic conditions (MSFBO), MSFBO clinical and dental features have not been compared to odontogenic sinusitis (ODS). This multicenter study aimed to compare characteristics of MSFB, MSFBO, and ODS.

**Methods:**

A multicenter international retrospective cohort study was conducted on adults with MSFBs and ODS who underwent sinus surgery. First in MSFBs, it was determined whether different dental conditions were more likely in FB versus non‐FB sides. Second, clinical features and dental pathologies were compared between MSFBO and ODS. For analyses, dental conditions were considered individually and as two groups: infectious pathologies and dental/oral procedures with indwelling metallic materials.

**Results:**

After exclusions, there were 203 MSFBs and 163 ODS. Among MSFBs, 141 were MSFBOs. FB sides were associated with sinus protrusion of root canal treatment (RCT) materials (*p* = 0.040) and dental implants (*p* = 0.040). Compared to MSFBO, ODS patients were younger, more likely to have MS purulence (OR = 40.9, *p* < 0.010), more likely associated with apical periodontitis (OR = 2.59, *p* = 0.010) and oroantral fistulas (OR = 6.94, *p* = 0.020), and less likely associated with extruded RCT materials (OR = 0.01, *p* = 0.010) and protruded midface screws (OR < 0.01, *p* = 0.010). Comparing purulent MSFBO and ODS, ODS was more associated with infectious dental pathologies (*p* < 0.009).

**Conclusion:**

Compared to MSFBs, MSFBOs were associated with RCT extrusion and implant protrusion. Compared to MSFBOs, ODS was more likely purulent and associated with infectious dental pathologies. While ODS is often distinct from MSFBO, the two conditions can coexist, and surgeons must determine whether patients have infectious dental pathology requiring treatment with both conditions.

**Level of Evidence:**

4.

## Graphical abstract

This international, multicenter study compared clinical and dental characteristics of maxillary sinus fungal balls (MSFB), odontogenic MSFB (MSFBO), and odontogenic sinusitis (ODS). When MSFB and MSFBO were compared, MSFBO was associated with extruded root canal material and dental implant protrusion; when MSFBOs and ODS were compared, ODS was associated with infectious dental conditions like apical periodontitis and oroantral fistulas. Surgeons should distinguish MSFB, MSFBO, and ODS, and determine whether concurrent dental pathology is infectious and treatable.

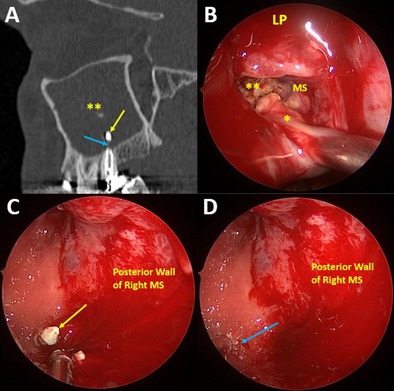

## 1 Introduction

Maxillary sinus fungal ball (MSFB) refers to a noninvasive extramucosal collection of fungal hyphae that may or may not be associated with bacterial infection [1], and can lead to invasive fungal sinusitis usually in the setting of immunocompromise [2, 3]. MSFBs can be associated with maxillary odontogenic or oral infections and procedures (MSFBO) [4, 5]. Odontogenic sinusitis (ODS) refers to bacterial infection of at least the maxillary sinus (MS), stemming from either infectious dental pathologies or complications following dental procedures [6]. MSFBO and ODS can share disease characteristics, and some clinicians may consider them to be similar pathophysiologically [7]. However, no studies have directly compared medical and dental features between MSFBO and ODS to explore their relationship.

Important reasons to distinguish ODS from MSFBO adjacent to dental conditions include being able to characterize more accurately the natural disease courses of each condition and to ensure optimal treatment options. One key point for clinicians to determine in these situations is whether patients have treatable infectious dental pathology. MSFBs generally require surgical removal via endoscopic sinus surgery (ESS) [1, 8], and if there is no dental pathology, no dental evaluation or treatment is necessary. When ODS is identified in the setting of treatable dental pathology, patients may resolve with definitive dental treatment alone or with ESS before or after the dental treatment [9, 10, 11, 12, 13]. If patients have MSFBOs, determining whether dental pathology is treatable dictates whether they need ESS or both ESS and dental treatment. To provide MSFB, MSFBO, and ODS patients with the most comprehensive diagnostic and therapeutic approach to both their dental and sinus pathologies, this study aimed to compare and contrast clinical features of the three disease states.

## 2 Methods

A retrospective cohort study was conducted on consecutive adult patients who had undergone ESS for unilateral MSFBs or ODS across 13 tertiary centers with subspecialty‐trained rhinologists between January 2016 and March 2025. Institutional Review Board approval was obtained at each participating site.

An a priori power analysis was performed to guide minimum sample size targets for comparing dental pathology prevalences in (1) MSFB patients between diseased and undiseased sides, and (2) MSFBO versus ODS (full details in Data S1). First, for comparing dental pathology prevalences in diseased versus nondiseased sides within MSFB patients, two published scenarios were utilized (2015 study [5]: π_1_ = 0.94, π_2_ = 0.84; 2022 study [4]: π_1_ = 0.20, π_2_ = 0.02). A two‐group chi‐square test with two‐sided α = 0.05 and 80% power was used for this analysis. The largest minimum sample size needed was 153 MSFBs. Second, to determine ODS and MSFBO sample sizes to compare dental pathology proportions between groups, the following proportions for dental pathologies were presumed: ODS without MSFB (π_1_ = 0.999) and MSFB without ODS (π_2_ = 0.94 [5], and 0.20 [4]). Using a two‐group chi‐square test for independent proportions with two‐sided α = 0.05 and 80% power, the largest required minimum sample size was 133 per group. To ensure adequate power across the prespecified questions, at least 133 ODS and 153 MSFB cases were targeted. Each center could provide up to 20 operative MSFB patients and up to their first 20 operative ODS patients during the time period of their recruited MSFB cohorts.

**FIGURE 1:**
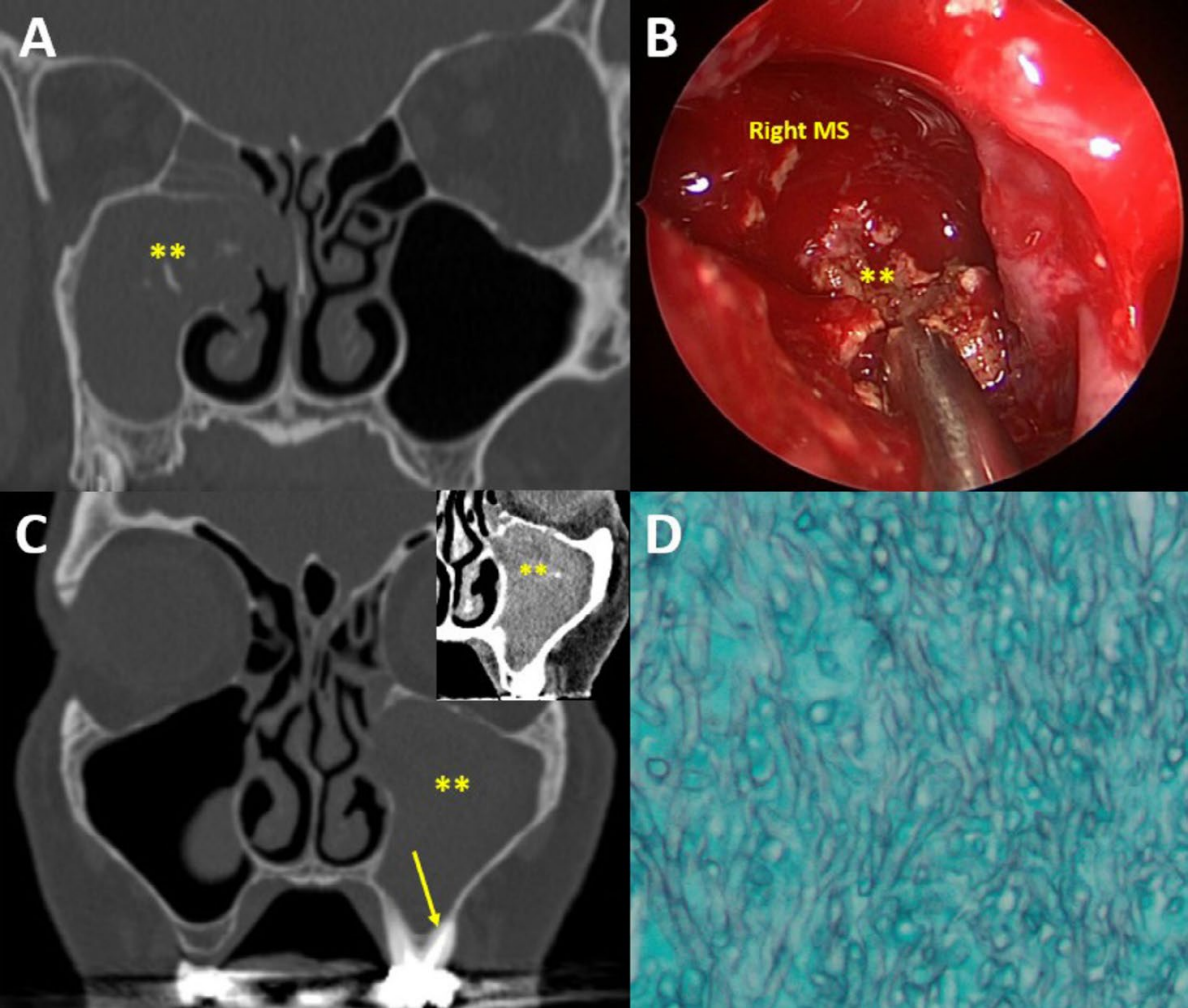
Two examples of maxillary sinus fungal balls without (A and B, MSFB) and with odontogenic conditions (C and D, MSFBO). (A) Coronal bone‐window CT showing right‐sided MSFB with intraluminal hyperdensities (double yellow asterisks). (B) 70° endoscopic view of the right maxillary sinus (MS) following maxillary antrostomy, showing the MSFB in the sinus (double yellow asterisks). (C) CT showing left maxillary sinus opacification (double yellow asterisks), but that only demonstrated intraluminal hyperdensities on soft‐tissue windowing (double yellow asterisks in upper right inserted image). (D) Grocott's methenamine silver staining for fungus of the left MSFBO, showing septate hyphae consistent with *Aspergillus* spp.

**FIGURE 2:**
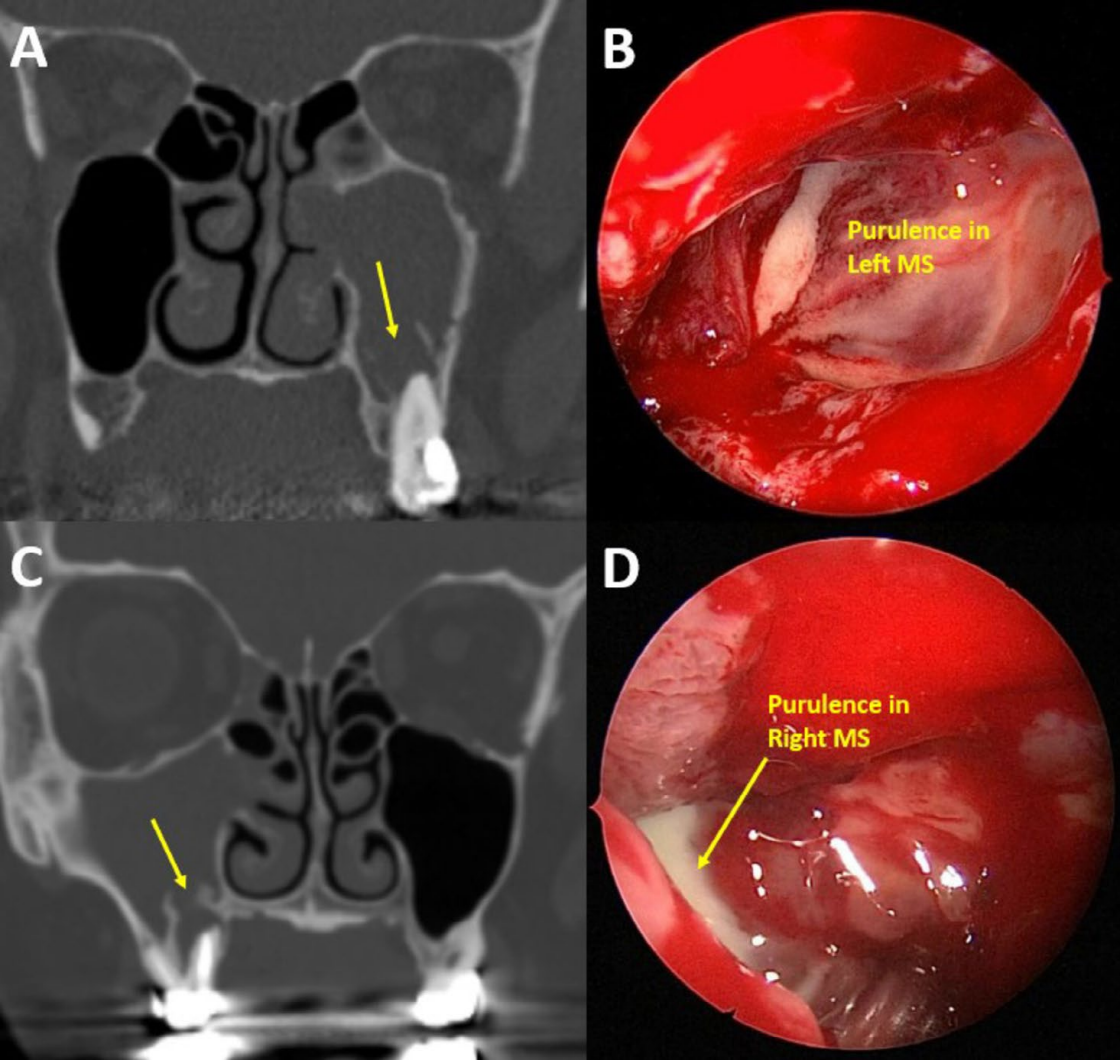
Two examples of odontogenic sinusitis (ODS) from apical periodontitis (A and B) and progressive periapical abscess following root canal treatment (C and D). (A) Coronal bone‐window CT showing left‐sided complete maxillary sinus opacification with an underlying molar with large periapical abscess with bone erosion (yellow arrow) in the setting of pulpal necrosis and apical periodontitis. (B) Left‐sided 70° endoscopic view of the left maxillary sinus (MS) following maxillary antrostomy, showing MS purulence. (C) CT showing right‐sided complete maxillary sinus opacification with an underlying molar with large periapical abscess with bone erosion (yellow arrow) following a prior root canal treatment. (D) 70° endoscopic view of the right maxillary sinus (MS) following maxillary antrostomy, showing MS purulence.

MSFB was defined as MS extramucosal fungal hyphae identified on Grocott's methenamine silver staining. MSFBO was defined as an MSFB with one or more associated dental or oral conditions seen on sinus CT (Figure 1; see dental conditions below). Diagnosing MSFBO did not necessarily require dental evaluations. ODS was defined as MS purulence confirmed endoscopically adjacent to confirmed maxillary dental conditions by dental specialists (Figure 2). Dental evaluation details like endodontic and periodontal examinations and imaging were not available for analysis.

The following demographic and clinical variables were collected: age, gender, ethnicity, and comorbidities (diabetes mellitus, current/former smoking history, long‐term corticosteroid use, primary immunodeficiency, HIV, and hemochromatosis). Other collected data for all patients included paranasal sinus opacification extent on CT, hyperdensities on CT for MSFBs, primary or revision surgery, and whether purulence was seen in MSs intraoperatively. CTs and electronic medical records were reviewed by fellowship‐trained rhinologists at each center to code dental pathologies and their respective tooth numbers. Before study onset, CT‐based examples of each dental pathology type were provided to authors (Data S2). Dental conditions were categorized by whether they represented infectious or possibly infectious pathologies (i.e., apical and marginal periodontitis, oroantral fistula on exam, and MS floor bone grafts with or without graft extrusion into sinus) or indwelling dental metallic materials (i.e., root canal treatments [RCT] with or without sinus extrusion, dental implants with or without MS protrusion, midface titanium screws with or without MS protrusion, freely floating metallic dental foreign bodies). For bone grafts and indwelling dental metallic materials, material extrusion or protrusion was determined by the relationship of the metallic elements to tooth apices for RCTs, and to the maxillary alveolar bone for bone grafts, implants, and screws. If materials were seen within the MS space on CT (mucosa or lumen), they were considered extruded or protruded. If uncertain of the origin of sinus hyperdensities in the setting of dental conditions, Hounsfield units of the intraluminal hyperdensities were measured and compared to the Hounsfield units of the dental materials (Figure S1) [14].

For both cohorts, patients were excluded if CT scans provided inadequate viewing of the maxillary dentition or dental materials, if they had MS opacification contralateral to the side of either MSFB or ODS, if fungal stain results were not reported, or if they had concurrent disease affecting the MSs that was not the MSFB or ODS (e.g., nasal polyps, neoplasia).

Various univariate and multivariate analyses (UVA, MVA) were conducted using R version 4.4.0. In UVAs, categorical variables were compared using McNemar's test, the Stuart–Maxwell test, or Fisher's exact test, as appropriate, while the continuous variable age was compared using a *t*‐test. *p* values were adjusted for multiple comparisons using the Benjamini–Hochberg procedure. In MVAs, generalized estimating equation (GEE) models with a logit link were applied to examine associations between clinical and dental features, accounting for within‐patient correlation since a single patient could contribute multiple dental pathologies. Other covariates were evaluated using separate generalized linear models with link functions selected according to the distribution of the outcome.

Within the MSFB cohort, demographic and clinical data were first compared between MSFB and MSFBO patients. Then FB and non‐FB sides were compared for the presence of the different maxillary dental conditions, both individually and by the following pathology groups: no dental pathology, infectious dental pathologies, and indwelling dental metallic elements. Next, demographic, clinical, and dental variables were compared between ODS and MSFBO patients. Dental pathologies were also compared between MSFBO patients with versus without MS purulence, and between those with ODS versus MSFBO with and without MS purulence. Variables were considered statistically significant if *p* < 0.05 on MVAs, or on UVA if no MVA was performed for certain comparisons.

## 3 Results

**FIGURE 3:**
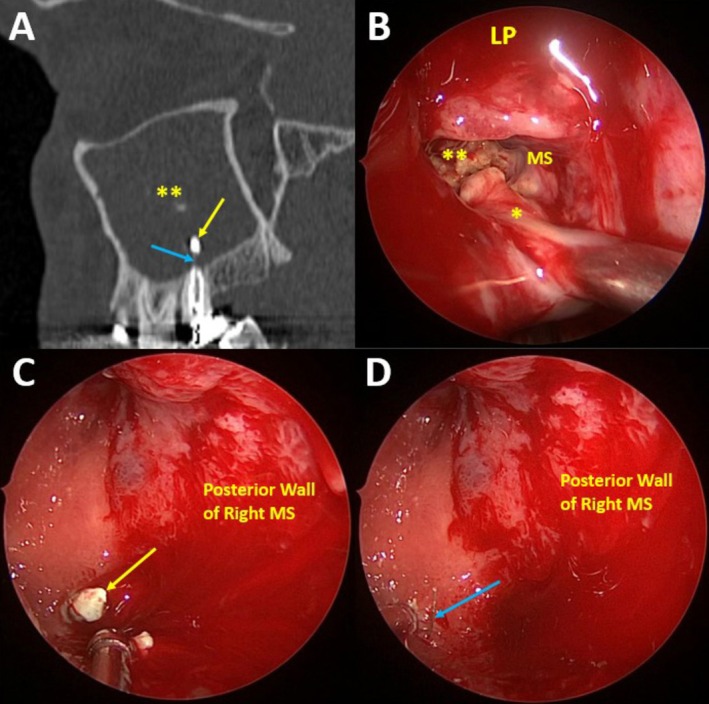
Example of a patient with both odontogenic sinusitis (ODS) and maxillary sinus fungal ball of odontogenic origin (MSFBO). The ODS was caused by a prior apical periodontitis and periapical abscess that was treated with a root canal treatment (RCT), after which the MSFBO developed. (A) Sagittal bone‐window CT showing complete right‐sided maxillary sinus opacification with intraluminal hyperdensities (double yellow asterisks), and an underlying molar with a RCT and extruded root canal materials (yellow arrow). Also note the faint hyperdense line between the tooth apex and extruded bright hyperdense material (blue arrow). (B) 70° endoscopic view of the right MS during the initial maxillary antrostomy, showing the MSFBO (double yellow asterisks) and associated purulence (single yellow asterisk). (C) 70° endoscopic view of the right MS following the completed modified endoscopic medial maxillectomy (MEMM), showing whitish debris attached to extruded metallic material emanating from the MS floor. (D) After removal of the whitish debris, a metal wire was seen emanating from the molars root apex (blue arrow), which was a separated endodontic instrument, most likely a stainless‐steel filer, and this was removed without creating an oroantral communication. The patient only required MEMM because the RCT was complete and viable.

Initially there were 239 MSFBs and 208 ODS patients. After inclusion and exclusion criteria were applied, analyses were performed with 203 MSFBs, 141 MSFBOs, and 163 ODS without MSFBs (Table S1). Note that 13/176 ODS patients had positive fungal staining for concurrent MSFBs (7.3%). Figure 3 shows an example of a patient with concurrent ODS and MSFB.

Table 1 shows demographic and clinical characteristics of MSFB and MSFBO patients across different regions internationally. Overall, 141/203 MSFBs had associated dental conditions, and therefore MSFBOs represented 69.5% of MSFBs. In the Polish cohort, all MSFBs had dental conditions (*p* < 0.010), whereas the other regions had no significant differences between MSFBO and MSFB rates. Within the USA, there were more MSFBOs in the Midwest (*p* = 0.010) and a nonsignificant association was seen in the West (*p* = 0.05). MSFBOs were less prevalent than MSFBs in the Northeastern and Southern USA (*p* < 0.010). Most other variables were not significantly different between MSFB and MSFBO, but former smoking was more likely in MSFBO (*p* = 0.020).

**TABLE 1:** Demographic and clinical variables compared between patients with maxillary sinus fungal balls (MSFB) and MSFB with odontogenic conditions (MSFBO). Bold *p* values were statistically significant.

Variables	MSFB (*n* = 62)	MSFBO (*n* = 141)	UVA *p* value	MVA OR	95% CI	MVA adjusted *p* value
Country (*n* [%])						
USA	41 (66.1)	86 (61.0)	0.590	0.45	(0.15, 1.02)	0.06
Italy	8 (12.9)	20 (14.2)	0.982	0.90	(0.26, 3.16)	0.85
Poland	0 (0.0%)	28 (19.9%)	**< 0.001**	8.25	(7.24, 11.70)	**< 0.01**
Taiwan	13 (21.0%)	7 (5.0%)	**0.001**	0.34	(< 0.01, 7.63)	0.44
USA regions (*n* [%])						
Northeast	11 (17.7%)	8 (5.7%)	**0.025**	0.14	(0.01, 0.35)	**0.01**
Midwest	1 (1.6%)	41 (29.1%)	**< 0.001**	31.50	(19.89, 47.47)	**< 0.01**
South	23 (37.1%)	11 (7.8%)	**< 0.001**	0.10	(0.02, 0.22)	**< 0.01**
West	6 (9.7%)	26 (18.4%)	0.124	3.48	(1.05, 27.71)	0.05
Age at surgery (years, mean [SD])	58.3 (15.0)	60.7 (13.2)	0.267	—	—	0.25
Male gender (*n* [%])	18 (29.0%)	49 (34.8%)	0.525	1.41	(0.69, 3.23)	0.35
Ethnicity (*n* [%])						
White	35 (56.5%)	97 (68.8%)	0.124	1.81	(0.87, 4.16)	0.12
Black	7 (11.3%)	6 (4.3%)	0.115	0.23	(0.01, 0.15)	0.06
Hispanic	2 (3.2%)	10 (7.1%)	0.452	2.34	(0.54, 25.86)	0.33
Asian	18 (29.0%)	24 (17.0%)	0.079	0.27	(0.27, 1.35)	0.24
Middle Eastern	0 (0.0%)	3 (2.1%)	0.599	—	—	—
Bermuda	0 (0.0%)	1 (0.7%)	1.000	—	—	—
Multinational, unknown	0 (0.0%)	0 (0.0%)	—	—	—	—
Comorbidities (*n* [%])						
Diabetes mellitus	12 (19.4%)	23 (16.3%)	0.744	0.84	(0.34, 2.17)	0.70
Current smoker	0 (0.0%)	6 (4.3%)	0.231	—	—	—
Former smoker	2 (3.2%)	25 (17.7%)	0.010	6.62	(2.08, 86.16)	**0.020**
Primary immunodeficiency	0 (0.0%)	0 (0.0%)	—	—	—	—
HIV	1 (1.6%)	0 (0.0%)	0.672	—	—	—
Chronic steroid use	0 (0.0%)	4 (2.8%)	0.429	—	—	—
Hemochromatosis	0 (0.0%)	0 (0.0%)	—	—	—	—
Right‐sided disease (*n* [%])	66 (40.5%)	67 (47.5%)	0.247	1.04	(0.53, 2.12)	0.91
Sinus opacification extent on CT (*n* [%])						
M only	26 (41.9%)	83 (58.9%)	**0.038**	1.44	(0.69, 3.17)	0.32
M, E	24 (38.7%)	32 (22.7%)	**0.029**	0.65	(0.32, 1.44)	0.25
M, E, F	9 (14.5%)	16 (11.3%)	0.688	0.96	(0.32, 2.80)	0.94
M, E, F, S	2 (3.2%)	7 (5.0%)	0.854	—	—	—
M, E, S	1 (1.6%)	3 (2.1%)	1.000	—	—	—
MS hyperdensities on CT (*n* [%])	60 (96.8)	128 (90.8)	0.309	0.35	(0.03, 1.10)	0.21
MS purulence (*n* [%])	40 (64.5%)	74 (52.5%)	0.150	0.74	(0.33, 1.46)	0.42
Primary surgery state (*n* [%])	54 (87.1%)	123 (87.2%)	1.000	0.76	(0.25, 2.48)	0.62

Abbreviations: CI, confidence interval; CT, computed tomography; M, maxillary; M, E, maxillary, ethmoid; M, E, F, maxillary, ethmoid, frontal; M, E, F, S, maxillary, ethmoid, frontal, sphenoid; M, E, S, maxillary, ethmoid, sphenoid; MS, maxillary sinus; MVA, multivariate analysis; UVA, univariate analysis.

Table 2 shows that among all MSFBs, non‐FB sides were more likely to have no dental pathology (*p* < 0.010) and prior dental extractions without OAFs (*p* = 0.040). When dental pathologies were analyzed as groups, only indwelling dental metallic elements showed a significant relationship with FB sides (*p* < 0.010; OR = 1.11, 95% CI: 1.03, 1.32), whereas infectious dental pathologies were not associated with FB sides (*p* = 0.680; OR = 1.02, 95% CI: 0.98, 1.09). On FB sides, prior RCTs with filling material extruding into sinuses (*p* = 0.040) and dental implants protruding into sinuses (*p* = 0.040) were significantly more likely on FB sides. When MSFBO patients were stratified by presence or absence of MS purulence, only post‐extractions without OAF were more likely in those without purulence (50.7% vs. 28.4%, *p* = 0.011). The other individual dental pathologies or groups occurred with similar frequencies between groups (Table S2).

**TABLE 2:** Comparisons of different dental pathologies and pathology groups between fungal ball (FB) and non‐FB sides in the whole MSFB cohort. Bold *p* values were statistically significant.

Dental pathologies in all MSFBs	FB side (*n* = 203)	Non‐FB side (*n* = 203)	Adjusted *p* value
No dental pathology (*n* [%])	62 (30.5%)	86 (42.4%)	**< 0.010**
Infectious or possible infectious dental pathologies (*n* [%])	91 (44.8)	94 (46.3)	0.680
Apical periodontitis with PAL	28 (13.8%)	19 (9.4%)	0.280
Postextraction without OAF	55 (27.1%)	72 (35.5%)	**0.040**
Postextraction with OAF	2 (1.0%)	0 (0.0%)	0.620
Marginal periodontitis	6 (3.0%)	2 (1.0%)	0.36
Bone graft without implant, without graft particle extrusion	0 (0.0%)	1 (0.5%)	1.000
Bone graft without implant, with graft particle extrusion	0 (0.0%)	0 (0.0%)	1.000
Indwelling dental metallic elements (*n* [%])	62 (30.5)	31 (15.3)	**< 0.010**
Prior RCT ± PAL, without filling material extruding into MS	23 (11.3%)	16 (7.9%)	0.360
Prior RCT ± PAL, with filling material extruding into MS	9 (4.4%)	0 (0.0%)	**0.040**
Dental implant ± bone graft, without protrusion into MS	10 (4.9%)	8 (3.9%)	0.810
Dental implant ± bone graft, with protrusion into MS	8 (3.9%)	0 (0.0%)	**0.040**
Midface screws without protrusion into MS	1 (0.5%)	3 (1.5%)	0.730
Midface screws with protrusion into MS	8 (3.9%)	4 (2.0%)	0.290
Free‐floating metallic dental foreign body in MS	3 (1.5%)	0 (0.0%)	0.360

Abbreviations: MS, maxillary sinus; OAF, oroantral fistula; PAL, periapical lesion; RCT, root canal treatment.

Table 3 shows demographic and clinical data compared via UVA and MVA between MSFBO and ODS without MSFB. ODS patients were younger than MSFBO patients (55.3 vs. 60.7 years, *p* = 0.0001), and were more likely to have MS purulence (100% vs. 52.5%, *p* < 0.001). MSFBO patients were more likely to have only MS opacification on CT, and while ODS patients had a higher rate of frontal in addition to maxillary and ethmoid sinus opacification on CT (36.2% vs. 11.3%), the association was not statistically significant (*p* = 0.05). Regarding dental conditions, ODS patients were more likely to have infectious or possibly infectious dental pathologies (85.9% vs. 63.1%, *p* < 0.001), and specifically apical periodontitis and OAFs (*p* < 0.001). MSFBO patients were more likely to have prior dental extractions without OAFs (39% vs. 16.6%, *p* < 0.001), as well as indwelling dental metallic elements (42.6% vs. 17.8%, *p* < 0.001). More specifically, MSFBO patients were more likely to have RCTs with and without filling materials extruding into sinuses (*p* < 0.027), and midface screws protruding into sinuses (*p* = 0.049).

**TABLE 3:** Demographic and clinical variables compared between patients with odontogenic sinusitis (ODS) and maxillary sinus fungal balls with odontogenic conditions (MSFBO). Bold *p* values were statistically significant.

Variables	ODS (*N* = 163)	MSFBO (*N* = 141)	UVA *p* value	MVA OR	95% CI	MVA *p* value
Country (*n* [%])						
USA	114 (69.9)	86 (61.0)	—	—	—	—
Italy	13 (8.0%)	20 (14.2%)	—	—	—	—
Taiwan	11 (6.7%)	28 (19.9%)	—	—	—	—
Poland	25 (15.3%)	7 (5.0%)	—	—	—	—
USA region (*n* [%])						
Northeast	25 (15.3%)	8 (5.7%)	—	—	—	—
Midwest	29 (17.8%)	41 (29.1%)	—	—	—	—
South	36 (22.1%)	11 (7.8%)	—	—	—	—
West	24 (14.7%)	26 (18.4%)	—	—	—	—
Age (years; mean [SD])	55.3 (16.0)	60.7 (13.2)	**0.001**	—	—	**< 0.010**
Male gender (*n* [%])	82 (50.3%)	49 (34.8%)	**0.008**	1.33	(0.64, 2.81)	0.420
Ethnicity (*n* [%])						
White	104 (63.8%)	97 (68.8%)	0.396	1.13	(0.53, 2.62)	0.750
Black	11 (6.7%)	6 (4.3%)	0.455	1.79	(0.32, 24.22)	0.530
Hispanic	4 (2.5%)	10 (7.1%)	0.061	0.17	(0.01, 2.24)	0.100
Asian	39 (23.9%)	24 (17.0%)	0.157	0.97	(0.38, 2.57)	0.950
Middle Eastern	2 (1.2%)	3 (2.1%)	0.666	—	—	—
Bermuda	1 (0.6%)	1 (0.7%)	1.000	—	—	—
Multinational, unknown	2 (1.2%)	0 (0.0%)	0.501	—	—	—
Comorbidities (*n* [%])						
Diabetes mellitus	19 (11.7%)	23 (16.3%)	0.200	0.65	(0.22, 1.69)	0.380
Current smoker	16 (9.8%)	6 (4.3%)	0.076	1.55	(0.28, 17.70)	0.560
Former smoker	28 (17.2%)	25 (17.7%)	0.914	2.18	(0.84, 7.23)	0.130
Primary immunodeficiency	1 (0.6%)	0 (0.0%)	1.000	—	—	—
HIV	2 (1.2%)	1 (0.7%)	1.000	—	—	—
Chronic PO steroids	4 (2.5%)	2 (1.4%)	0.689	—	—	—
Hemochromatosis	0 (0.0%)	0 (0.0%)	—	—	—	—
Right‐sided disease (*n* [%])	66 (40.5%)	67 (47.5%)	0.247	0.60	(0.14, 2.07)	0.420
Sinus opacification extent on CT (*n* [%])						
M only	50 (30.7%)	83 (58.9%)	**< 0.001**	0.49	(0.21, 0.91)	**0.040**
M, E	38 (23.3%)	32 (22.7%)	1.000	0.84	(0.36, 1.95)	0.660
M, E, F	59 (36.2%)	16 (11.3%)	**< 0.001**	2.19	(1.00, 6.27)	0.050
M, E, F, S	16 (9.8%)	7 (5.0%)	0.131	1.97	(0.70, 9.62)	0.280
M, E, S	0 (0.0%)	3 (2.1%)	0.099	—	—	—
MS purulence (*n* [%])	163 (100.0%)	74 (52.5%)	**< 0.001**	40.9	(4.01, 190.60)	< 0.010
Primary surgery state (*n* [%])	150 (92.0%)	123 (87.2%)	0.187	0.57	(0.13, 1.97)	0.370
Infectious or possibly infectious dental pathologies (*n* [%])	140 (85.9%)	89 (63.1%)	**< 0.001**	7.39	(4.39, 28.41)	**< 0.010**
Apical periodontitis with PAL	72 (44.2%)	28 (19.9%)	**< 0.001**	2.59	(1.41, 6.35)	**0.010**
Postextraction without OAF	27 (16.6%)	55 (39.0%)	**< 0.001**	0.73	(0.31, 1.56)	0.470
Postextraction with OAF	33 (20.2%)	2 (1.4%)	**< 0.001**	6.94	(2.31, 164.94)	**0.020**
Marginal periodontitis	5 (3.1%)	4 (2.8%)	1.000	—	—	—
Bone graft without implant, without graft particle extrusion	5 (3.1%)	0 (0.0%)	0.064	—	—	—
Bone graft without implant, with graft particle extrusion	1 (0.6%)	0 (0.0%)	1.000	—	—	—
Indwelling dental metallic elements (*n* [%])	29 (17.8%)	59 (41.8%)	**< 0.001**	0.16	(0.05, 0.29)	**< 0.010**
Prior RCT ± PAL, without filling material extruding into MS	10 (6.1%)	23 (16.3%)	**0.005**	0.20	(0.04, 0.49)	**< 0.010**
Prior RCT ± PAL, with filling material extruding into MS	2 (1.2%)	9 (6.4%)	**0.027**	0.01	(< 0.01, 0.09)	**0.010**
Dental implant ± bone graft, without protrusion into MS	5 (3.1%)	10 (7.1%)	0.126	0.26	(0.01, 1.40)	0.100
Dental implant ± bone graft, with protrusion into MS	9 (5.5%)	8 (5.7%)	1.000	0.56	(0.09, 2.30)	0.390
Midface screws without protrusion into MS	0 (0.0%)	1 (0.7%)	1.000	—	—	—
Midface screws with protrusion into MS	2 (1.2%)	8 (5.7%)	**0.049**	< 0.01	(< 0.01, < 0.01)	**0.010**
Free‐floating metallic dental foreign body in MS	1 (0.6%)	3 (2.1%)	0.340	—	—	—

Abbreviations: CI, confidence interval; CT, computed tomography; M, maxillary; M, E, maxillary, ethmoid; M, E, F, maxillary, ethmoid, frontal; M, E, F, S, maxillary, ethmoid, frontal, sphenoid; M, E, S, maxillary, ethmoid, sphenoid; MS, maxillary sinus; MVA, multivariate analysis; UVA, univariate analysis.

Table 4 shows comparisons of dental pathology types between ODS and both MSFBO with and without MS purulence. For ODS versus both MSFBO with and without MS purulence, infectious dental pathologies were significantly more likely in ODS, specifically with apical periodontitis and OAFs after extractions. On the contrary, indwelling metallic dental materials were significantly more likely in MSFBOs than ODS, both for MSFBO with and without MS purulence. Finally, of note, relative to ODS, RCT filling extrusion was more likely in MSFBO without MS purulence (*p* = 0.012), whereas RCT filling without extrusion was more likely in MSFBO with purulence (*p* = 0.005).

**TABLE 4:** Comparisons of different dental pathologies and pathology groups between odontogenic sinusitis (ODS) and maxillary sinus fungal balls with odontogenic conditions (MSFBO) both with and without maxillary sinus (MS) purulence. Bold *p* values were statistically significant.

Dental pathologies in ODS and MSFBOs	ODS (*n* = 163)	MSFBO with MS purulence (*n* = 74)	*p* value (ODS vs. MSFBO with purulence)	MSFBO without MS purulence (*N* = 67)	*p* value (ODS vs. MSFBO without purulence)
No dental pathology (*n* [%])	0 (0.0%)	0 (0.0%)	—	0 (0.0%)	—
Infectious or possibly infectious dental pathologies (*n* [%])	140 (85.9%)	42 (56.8%)	**< 0.001**	47 (70.1%)	**0.009**
Apical periodontitis with PAL	72 (44.2%)	16 (21.6%)	**0.001**	12 (17.9%)	**< 0.001**
Postextraction without OAF	27 (16.6%)	21 (28.4%)	0.055	34 (50.7%)	**< 0.001**
Postextraction with OAF	33 (20.2%)	2 (2.7%)	**0.001**	0 (0.0%)	**< 0.001**
Marginal periodontitis	3 (1.8%)	3 (4.1%)	0.576	3 (4.5%)	0.493
Bone graft without implant, without graft particle extrusion	5 (3.1%)	0 (0.0%)	0.301	0 (0.0%)	0.341
Bone graft without implant, with graft particle extrusion	1 (0.6%)	0 (0.0%)	1.000	0 (0.0%)	1.000
Indwelling dental metallic materials (*n* [%])	29 (17.8%)	35 (47.3%)	**< 0.001**	24 (35.8%)	**0.005**
Prior RCT ± PAL, without filling material extruding into MS	10 (6.1%)	14 (18.9%)	**0.005**	9 (13.4%)	0.118
Prior RCT ± PAL, with filling material extruding into MS	2 (1.2%)	3 (4.1%)	0.360	6 (9.0%)	**0.012**
Dental implant ± bone graft, without protrusion into MS	5 (3.1%)	6 (8.1%)	0.169	3 (4.5%)	0.893
Dental implant ± bone graft, with protrusion into MS	9 (5.5%)	6 (8.1%)	0.638	2 (3.0%)	0.632
Midface screws without protrusion into MS	0 (0.0%)	1 (1.4%)	0.685	0 (0.0%)	—
Midface screws with protrusion into MS	2 (1.2%)	4 (5.4%)	0.147	4 (6.0%)	0.111
Free‐floating metallic dental foreign body in MS	1 (0.6%)	1 (1.4%)	1.000	2 (3.0%)	0.423

Abbreviations: OAF, oroantral fistula; PAL, periapical lesion; RCT, root canal treatment.

## 4 Discussion

MSFB is a relatively common cause of MS, and while it can remain asymptomatic without complications, it can also lead to invasive fungal sinusitis in immunodeficient patients [2, 3]. MSFBs can develop after fungal spore inhalation transnasally or possibly via transoral migration from infected or colonized dentition or dental hardware, with *Aspergillus* spp. being the most common causative fungi [15, 16, 17]. Various factors have been associated with MSFB development, with dental implants and RCTs being the most commonly associated dental conditions [4, 5, 18]. Additionally, some studies have also reported infectious dental pathologies being associated with MSFBs, like dental extractions and apical periodontitis [4, 18].

Based on prior international consensus, ODS was defined as bacterial sinusitis adjacent to dental or oral bacterial infectious conditions, while fungal etiologies were omitted [6]. A recent review and consensus article reported that MSFBs may coexist with ODS [19], while others have considered both bacterial and fungal sinusitis associated with dental conditions to fall under the ODS umbrella [7]. Prior studies on MSFBOs have not reported the frequency of concurrent bacterial sinusitis, nor have they compared clinical findings directly to ODS patients. Therefore, it has been difficult to know whether MSFBO and ODS are distinct conditions or how often they coexist. The current study built on prior studies with a powered analysis of ODS and MSFB patients through a multicenter international collaboration to improve generalizability across otolaryngologists' practices.

In the current study, dental conditions were seen in about 70% of MSFBs, with FB sides in MSFBOs being significantly associated with prior dental procedures with metallic materials. While prior studies have also shown this, the current study showed more specifically that extruded RCT or protruding dental implant materials were associated with MSFBOs. This is in line with prior literature showing *Aspergillus* spp. having an affinity for RCT filling materials like Zinc‐oxide [20] and metallic dental implants [21]. Notably, this is the first study suggesting that dental metallic elements contained within the dentition or oral side of the sinus floor may not be associated with MSFB development. Another interesting finding was that in MSFBO patients without ODS, there were no significant differences in infectious dental pathologies or indwelling dental material between those with or without MS purulence. This could imply that at least some of these purulent MSFBO cases were sinogenic bacterial superinfections rather than odontogenic. Future studies utilizing bacterial and fungal cultures or next‐generation sequencing would be beneficial in determining the most likely source of purulence in MSFBOs.

Comparing clinical features of ODS and MSFBO patients, the most glaring differences between the groups were the rates of MS purulence and associated dental pathologies. All ODS patients had MS purulence compared to about half of MSFBO patients. Regarding dental conditions, ODS patients were significantly more likely to have infectious dental pathologies like apical periodontitis and OAFs, whereas MSFBO patients were more likely to harbor indwelling dental metallic materials like RCTs and titanium screw protrusion. When further stratified by the presence of MS purulence, apical periodontitis and OAFs remained significantly associated with ODS compared to MSFBOs. While there was no difference in rates of dental implants between ODS and MSFBO, it is possible the sample sizes of these conditions were too small to detect a difference.

This study would support the notion that ODS is often distinct from MSFBO in that ODS was universally purulent, most commonly stemmed from infectious dental pathology, and only 7% of ODS patients had concurrent MSFBs. However, it is also important to note that among the MSFBO patients with MS purulence, some occurred with infectious dental pathologies (e.g., 22% had apical periodontitis). Therefore, some of the MSFBO patients with infectious dental pathologies either had MSFBs with concurrent ODS, or the MSFBOs truly stemmed from the infectious dental pathologies. Future studies should employ CT and nasal endoscopy before and after dental interventions to explore the temporal relationship between dental treatments and MSFB development. Ideally bacterial and fungal cultures would be collected as well to determine the most likely sources of these organisms. Conducting such studies prospectively with multidisciplinary collaboration would be difficult, but one way to determine more precisely the origins of ODS and MSFBO.

More critical than whether MSFBO and ODS represent distinct entities is that otolaryngologists manage the sinusitis optimally, and determine whether possible dental sources are infectious and treatable. Based on this study's findings, if patients have an isolated MSFBO and no MS purulence, it is significantly less likely that they will have infectious dental pathology requiring treatment. However, when MS purulence is seen in the setting of MSFB or MSFBO, surgeons should consider whether patients have infectious dental disease warranting evaluation. Dental pathology will usually be evident on sinus CT (e.g., periapical lesions, OAC/OAF, implants or bone grafts with bone erosion) [22, 23], but in the absence of overt dental pathology on CT, surgeons may not be able to know for certain whether an underlying dental condition is an infectious source. These patients could be referred to dental specialists for determination, or they could be monitored for persistent or recurrent MS disease following ESS with serial nasal endoscopy. Future studies are needed to determine whether some MSFBO patients are at higher risk for failure after ESS, but until then, surgeons must use judgment as to who warrants dental evaluations.

Multiple study limitations should be discussed. First, as dental evaluation details were not analyzed in this study, one must acknowledge potential inaccuracies of diagnosing certain dental pathologies based on sinus CT alone. This would mainly have confounded those with MSFBOs, since dental evaluations were not mandatory for these. Another related limitation was that rhinologists were not blinded on CT review, and only single reviewers were employed, which prevented interrater agreement calculations. While most of the dental pathologies were readily identifiable on CT, and all authors were provided prestudy CT examples of each dental pathology type, the methodology was imperfect for the most precise and reliable identification of dental pathologies. With ODS, there was confounding with diagnosing apical versus marginal periodontitis due to the lack of dental testing information. Endodontic testing is necessary to determine whether periodontal plus periapical bone erosion on CT is due to an endodontic or periodontal infectious origin. While this should not have affected this study's findings since both apical and marginal periodontitis were grouped under infectious dental conditions, it is possible that marginal periodontitis was under‐represented in this study. Another issue with lacking dental evaluations for MSFBOs was that it was impossible to determine precisely what proportion of MSFBO patients with MS purulence had ODS, and whether those patients had infectious dental pathology requiring treatment. Additionally, due to the retrospective design, it cannot be determined whether the dental conditions in those with MSFBOs actually caused the MSFB. Importantly, however, this large multi‐institutional and international study showed that MSFBOs and ODS were often associated with different types of dental sources, and that they can coexist. Lastly, while this study included centers from different regions of the world, it did not include low‐income countries with reduced access to CT imaging and certain dental procedures like RCT and dental implants. The ratio of MSFBO to ODS could be different in those countries, which could limit the utility of this study's findings for providers in such countries. However, it is still beneficial for clinicians in all areas worldwide to be aware that ODS and MSFBO exist, and may coexist.

## 5 Conclusions

Compared to MSFBs, MSFBOs were associated with RCT filling extrusion and dental implant protrusion. Compared to MSFBOs, ODS was more likely purulent and associated with infectious dental pathologies. While ODS is often distinct from MSFBO, the two conditions can coexist, and surgeons must determine whether patients have treatable dental pathology with both conditions.

## Funding

The authors have nothing to report.

## Conflicts of Interest

John R. Craig: Research consultant for Aerin Medical Inc. The other authors declare no conflicts of interest.

## Supporting information


**Figure S1:** Coronal bone‐window CT showing a bright hyperdensity in the completely opacified right maxillary sinus which was considered to be extruded root canal material based on matching Hounsfield units (3071) between the sinus hyperdensity and the hyperdense filling material within the underlying molar's pulp chamber.

**Data S1:** Details of the power analysis performed by statistician (Jun Jin, PhD).

**Data S2:** Examples of different dental pathologies and how to code them, plus how to determine whether intraluminal hyperdensities are likely from dental conditions using Hounsfield units.

**Table S1:** Reasons for excluding certain patients from maxillary sinus fungal ball (MSFB) and odontogenic sinusitis (ODS) cohorts.

**Table S2:** Comparisons of different dental pathologies and pathology groups between maxillary sinus fungal balls with odontogenic conditions (MSFBO) with versus without maxillary sinus (MS) purulence. MS, maxillary sinus; OAF, oroantral fistula; PAL, periapical lesion; RCT, root canal treatment. Bold *p* values bolded were statistically significant.

## Data Availability

The data that support the findings of this study are available from the corresponding author upon reasonable request.
