# Supplementary figures and images for: Clinical Features and Dental Pathologies in Maxillary Sinus Fungal Balls and Odontogenic Sinusitis
Source: Laryngoscope. 2026 Feb 7;136(7):2913–24. doi: 10.1002/lary.70429 (PMC13253162; doi:10.1002/lary.70429)

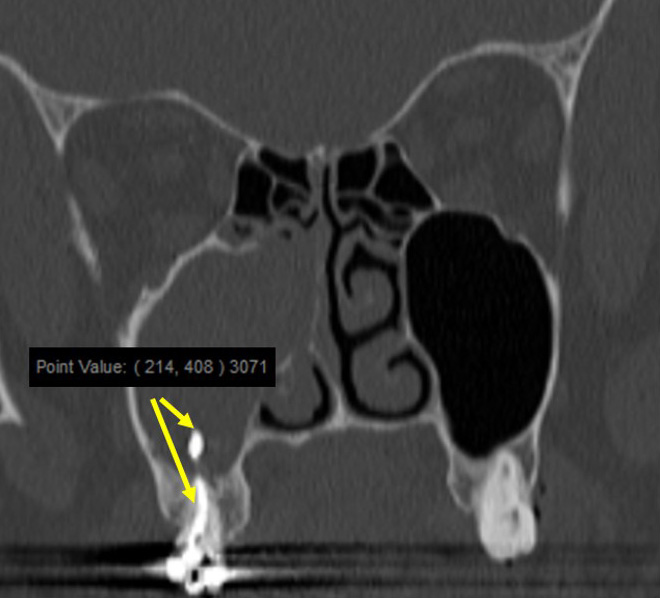

Supplement: Supplementary file 1 — Figure S1: Coronal bone‐window CT showing a bright hyperdensity in the completely opacified right maxillary sinus which was considered to be extruded root canal material based on matching Hounsfield units (3071) between the sinus hyperdensity and the hyperdense filling material within the underlying molar's pulp chamber. [file LARY-136-2913-s001.jpg]
