# Supplementary material for: Clinical Features and Dental Pathologies in Maxillary Sinus Fungal Balls and Odontogenic Sinusitis
Source: Laryngoscope. 2026 Feb 7;136(7):2913–24. doi: 10.1002/lary.70429 (PMC13253162; doi:10.1002/lary.70429)
Supplement: Supplementary file 2 — Data S1: Details of the power analysis performed by statistician (Jun Jin, PhD). [file LARY-136-2913-s004.docx]

- Question 1 for power analysis:

**Hypotheses**: that the proportions of identifiable odontogenic dental pathologies differs between fungal ball and healthy sides in patients with MSFBs.

**Primary endpoint:** the proportion of patients (π₁ and π₂) with identifiable dental pathologies when comparing fungal ball (diseased) to diseased sides within MSFB patients.

**Significance level:** two-sided α = 0.05

**Power:** 80%

**Sample-size calculation method:** we used a conservative scenario based on published data (2015 and 2022 studies), assuming π₁ = 0.94 (using 2015 study) or π₁ = 0.20 (using 2022 study) in MSFB fungus ball side and π₂ = 0.84 (using 2015 study) or π₂ = 0.02 (using 2022 study) in the healthy side (absolute difference 10% or 18% as the effect size, or say OR ≈ 0.335 or 0.082, using Ψ = π₂(1−π₁) / [π₁(1−π₂)]), evaluated using a two-group χ² test, with two-sided α = 0.05 and 80% power, yielding a required sample size of 153 (using 2015 study) or 47 (using 2022 study) MSFB patients. **So we take 153 as the required patient sample sizes for the MSFB cohort in this question.** The output from R is shown below.

**Software:** R version 4.4.0


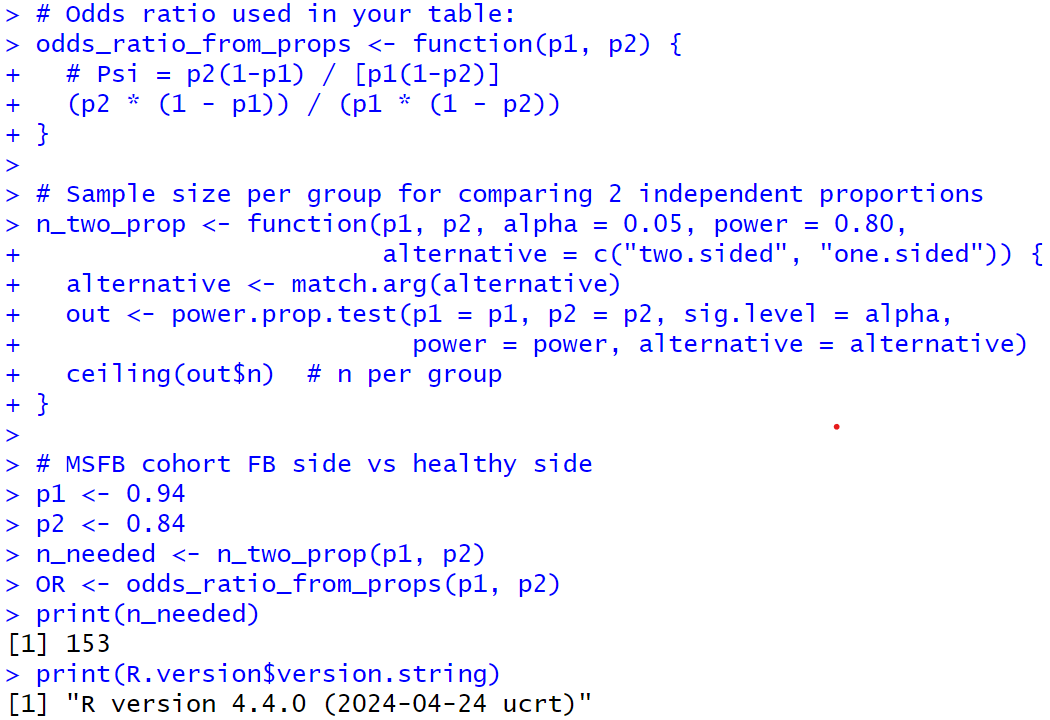
(2015 study result)


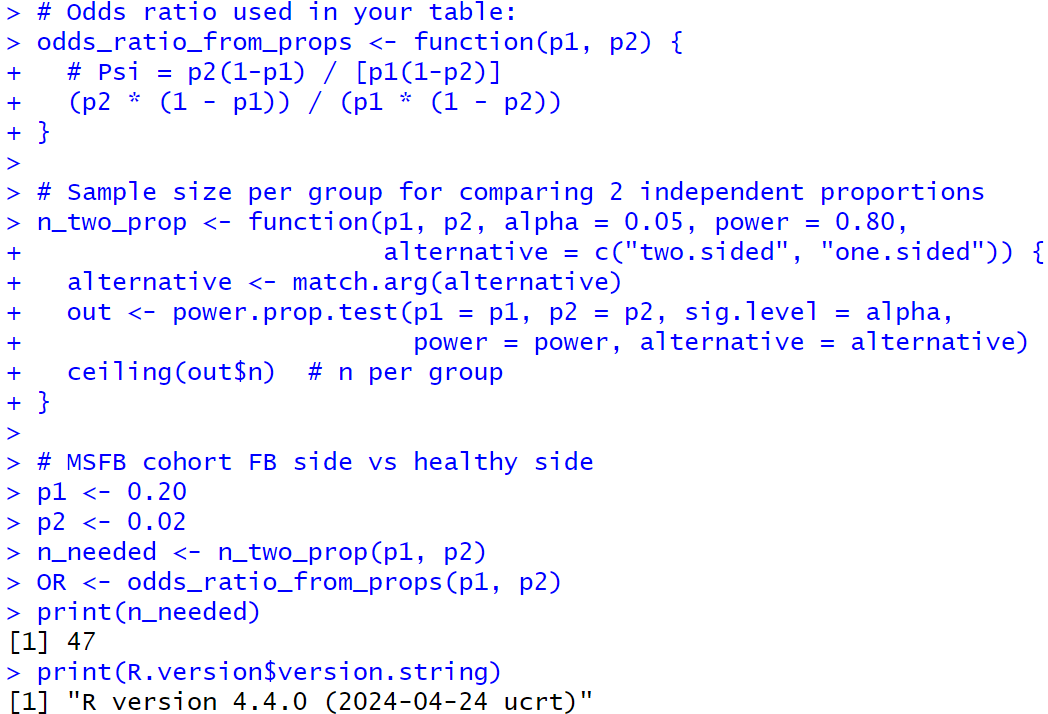
(2022 study result)

|  | Dental pathology proportions in FBs versus healthy sides  (2022 Study) | Dental pathology proportions in FBs versus healthy sides  (2015 Study) |
| --- | --- | --- |
| Test Significance Level, α​ | 0.05 | 0.05 |
| 1 or 2 Sided Test?​ | Two | Two |
| Group 1 Proportion, π₁​ | 0.2 | 0.94 |
| Group 2 Proportion, π₂​ | 0.02 | 0.84 |
| Odds Ratio, Ψ=π₂(1-π₁)/(π₁(1-π₂))​ | 0.081632653 | 0.335106383 |
| Power (%)​ | 80 | 80 |
| Sample Size per Group, n​ | 47 | 153 |

- Question 2 for power analysis:

**Hypotheses**: that the proportions of identifiable odontogenic dental pathologies differs between ODS and MSFBO cohorts.

**Primary endpoint:** the proportion of patients (π₁ and π₂) with identifiable dental pathologies when comparing the ODS (no fungus) versus MSFB (no ODS) cohorts.

**Significance level:** two-sided α = 0.05

**Power:** 80%

**Sample-size calculation method:** we used a conservative scenario based on published data (2015 and 2022 studies), assuming π₁ = 0.999 in ODS and π₂ = 0.94 (using 2015 study) or π₂ = 0.20 (using 2022 study) in MSFB (absolute difference 5.9% or 79.9% as the effect size, or say OR ≈ 0.016 or 0.0003, using Ψ = π₂(1−π₁) / [π₁(1−π₂)]), evaluated using a two-group χ² test, with two-sided α = 0.05 and 80% power, yielding a required sample size of 133 (using 2015 study) or 5 (using 2022 study) per group. **So, we take 133 as the required patient sample sizes for both ODS and MSFD cohorts to answer this question.** The output from R is shown below.

**Software:** R version 4.4.0


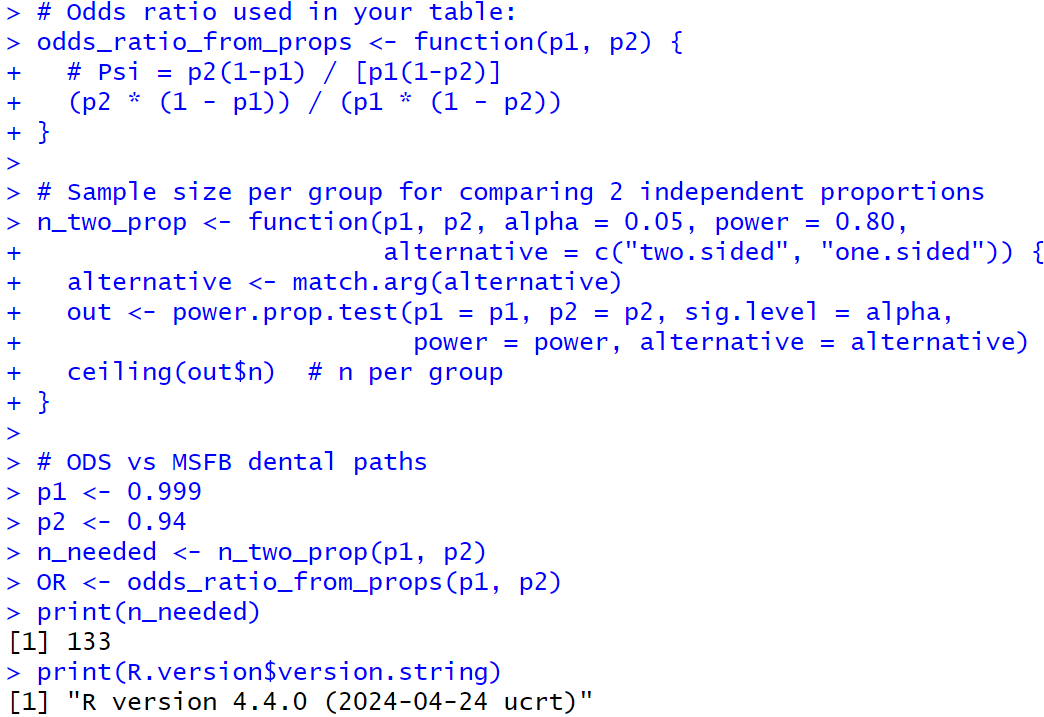
 (2015 study result)


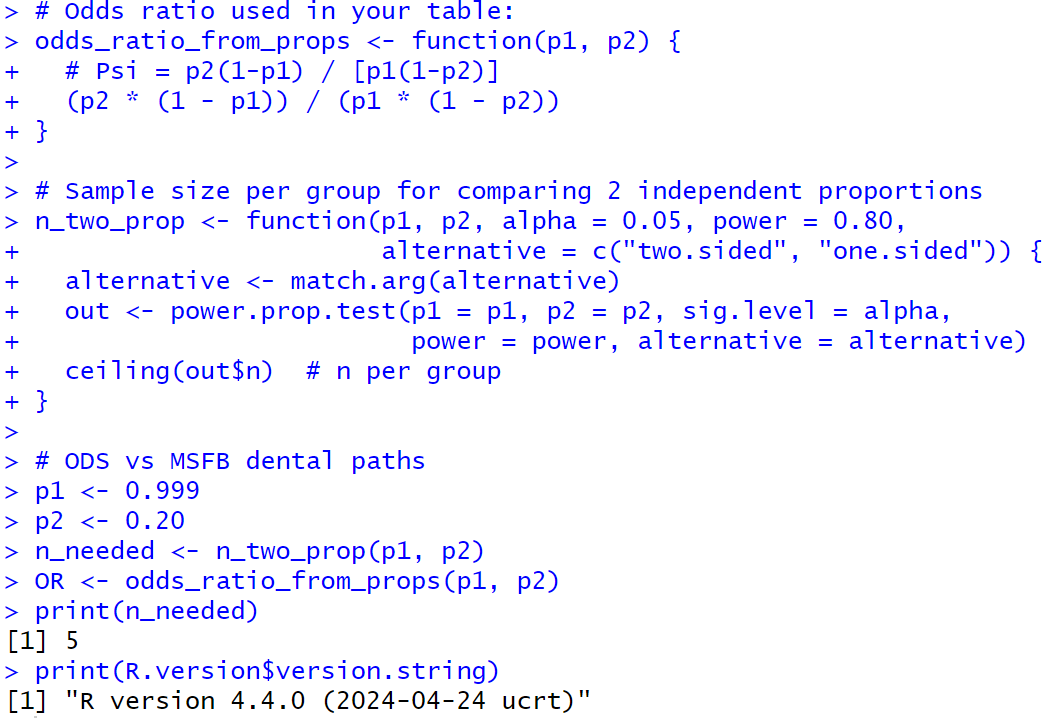
(2022 study result)

|  | ODS (no MSFB) with Dental Pathologies (100%) versus MSFB (no ODS) with Dental Pathologies (2022 study) | ODS (no MSFB) with Dental Pathologies (100%) versus MSFB (no ODS) with Dental Pathologies (2015 study) |
| --- | --- | --- |
| Test Significance Level, α​ | 0.05 | 0.05 |
| 1 or 2 Sided Test?​ | Two | Two |
| Group 1 Proportion, π₁​ | 0.999 | 0.999 |
| Group 2 Proportion, π₂​ | 0.2 | 0.94 |
| Odds Ratio, Ψ=π₂(1-π₁)/(π₁(1-π₂))​ | 0.0003 | 0.0157 |
| Power (%)​ | 80 | 80 |
| Sample Size per Group, n​ | 5 | 133 |

- Summary: Based on all above, we take 133 as the required patient sample size for ODS cohort and 153 as the required patient sample size for MSFB cohort.
