# Supplementary material for: Clinical Features and Dental Pathologies in Maxillary Sinus Fungal Balls and Odontogenic Sinusitis
Source: Laryngoscope. 2026 Feb 7;136(7):2913–24. doi: 10.1002/lary.70429 (PMC13253162; doi:10.1002/lary.70429)

Dental Paths and Tooth Numbering from Excel

**Dental Paths (no indwelling dental treatment materials)**	
0	None
1	Apical periodontitis (+/- periapical lesion on imaging)
2	Post-extraction without OAF on exam (make note if suspected alveolar bone or tooth fragments in sinus, if you can tell)
3	Post-extraction with OAF on exam (make note if suspected alveolar bone or tooth fragments in sinus, if you can tell)
4	Marginal periodontitis (negative endodontic testing ideally)
5	Prior bone graft but no implant, without graft particle extrusion into sinus lumen
6	Prior bone graft but no implant, WITH graft particle extrusion into sinus lumen (if you can determine)
**Dental Paths (dental treatments with treatment materials)**	
7	Prior root canal +/- periapical lesion on imaging (WITHOUT extruded root canal filling into the maxillary sinus lumen)
8	Prior root canal +/- periapical lesion on imaging (WITH extruded root canal filling into the maxillary sinus lumen)
9	Dental implant (+/- bone graft) NOT protruding into maxillary sinus lumen (any amount of implant not cover by bone and abutting sinus)
10	Dental implant (+/- bone graft) WITH protrusion into maxillary sinus lumen (any amount of implant not cover by bone and abutting sinus)
11	Midface plates of screws NOT protruding into maxillary sinus lumen (any amount of implant not cover by bone and abutting sinus)
12	Midface plates WITH protrusion into maxillary sinus lumen (any amount of hardware not cover by bone and abutting sinus)
13	Metallic dental foreign body freely floating in maxillary sinus lumen (ie NOT attached to alveolar ridge or maxilla)
**Tooth numbers (1-16; use AXIAL CT to count out teeth)**	
Right maxillary last molar= 1	
Left maxillary last molar= 16	
Edentulous= 17	

Dental path 0 (none)

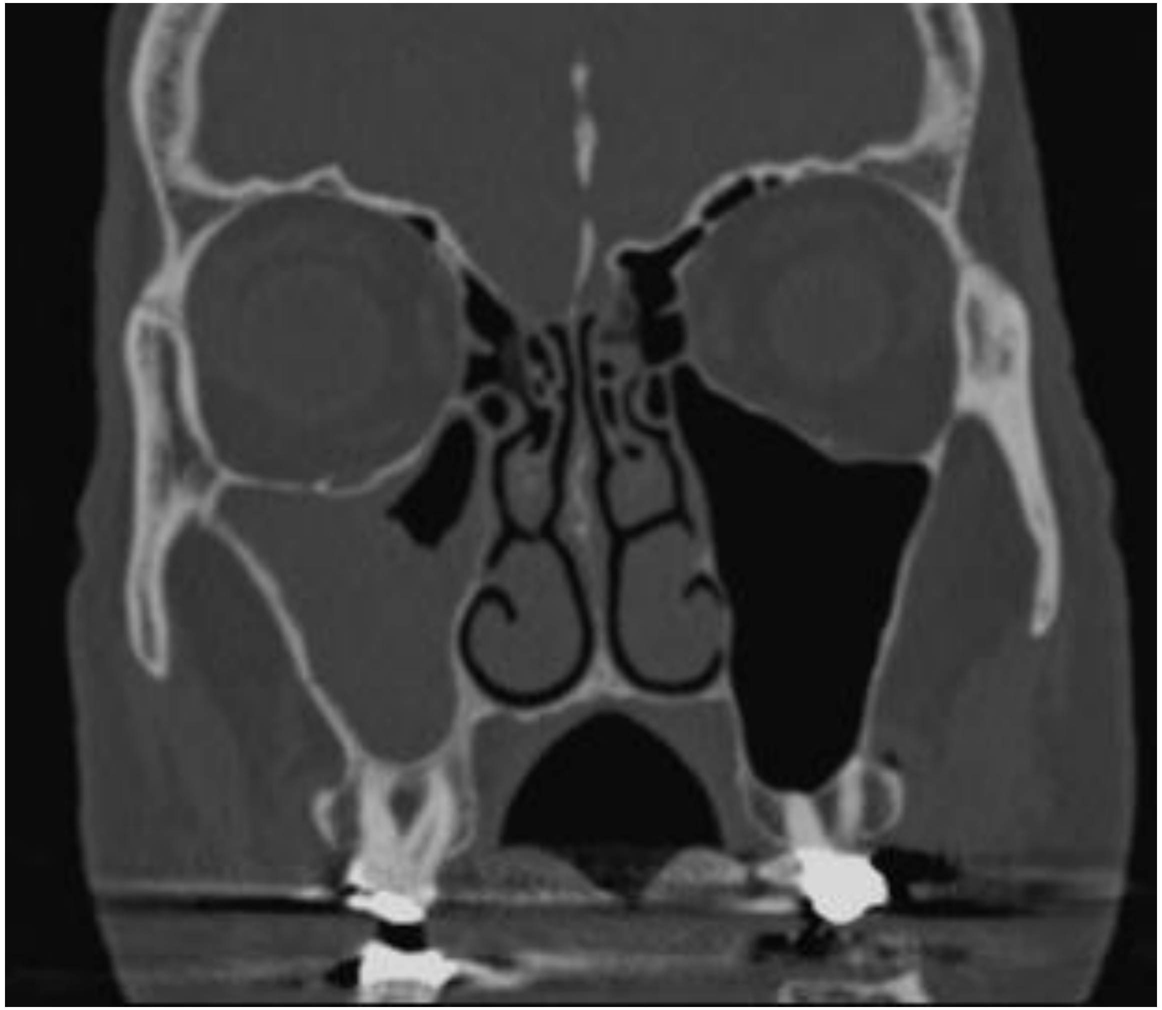

Path 1, tooth 15 (axial is most reliable for tooth numbering; can use sagittal to help)

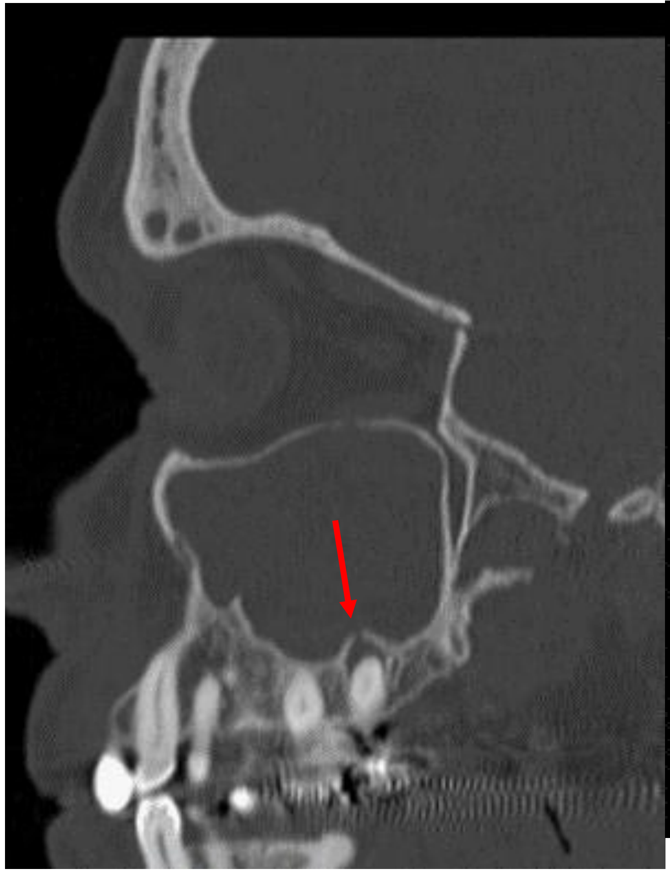

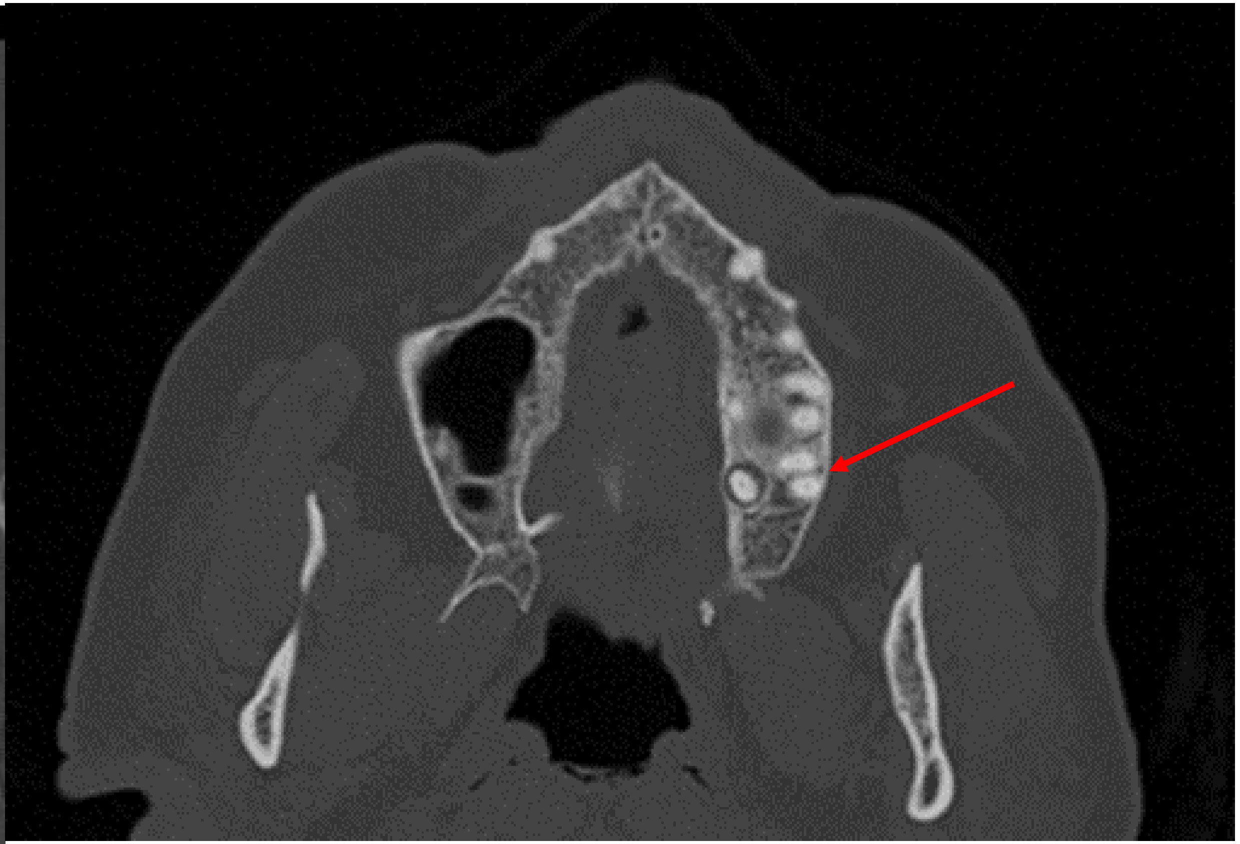

Coding as a 2 for tooth #16 in this case

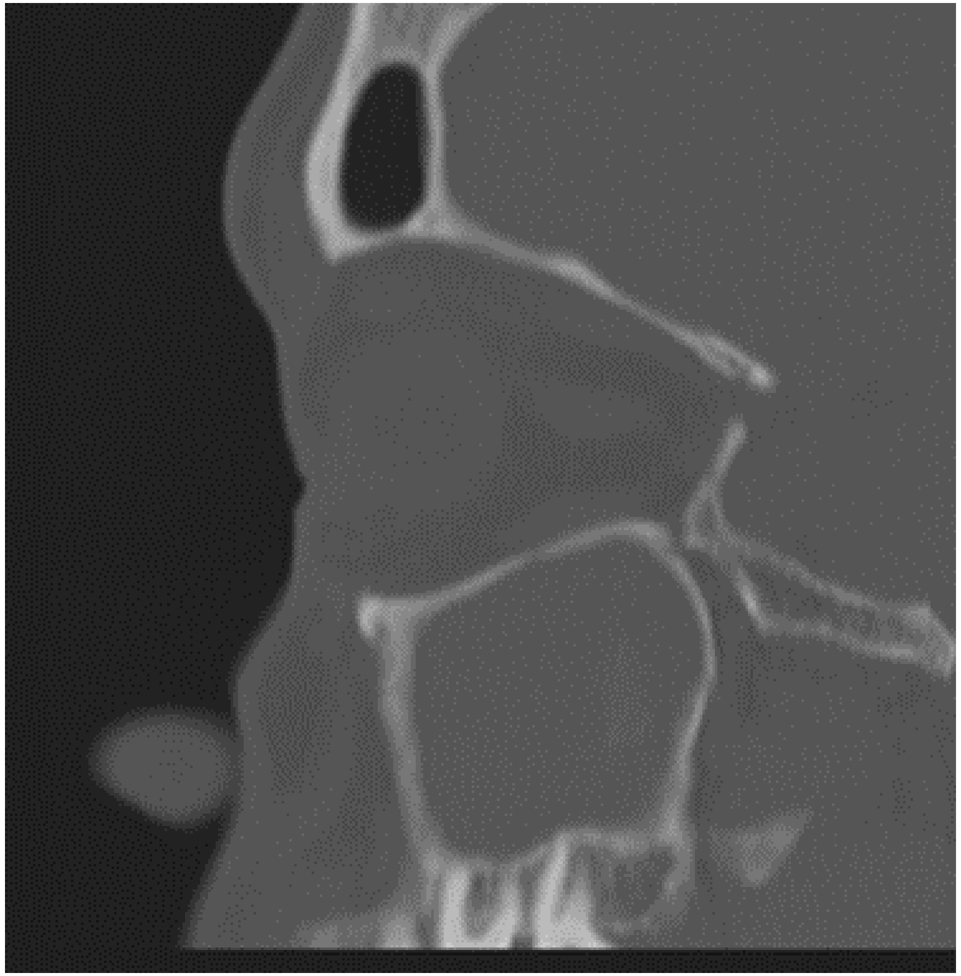

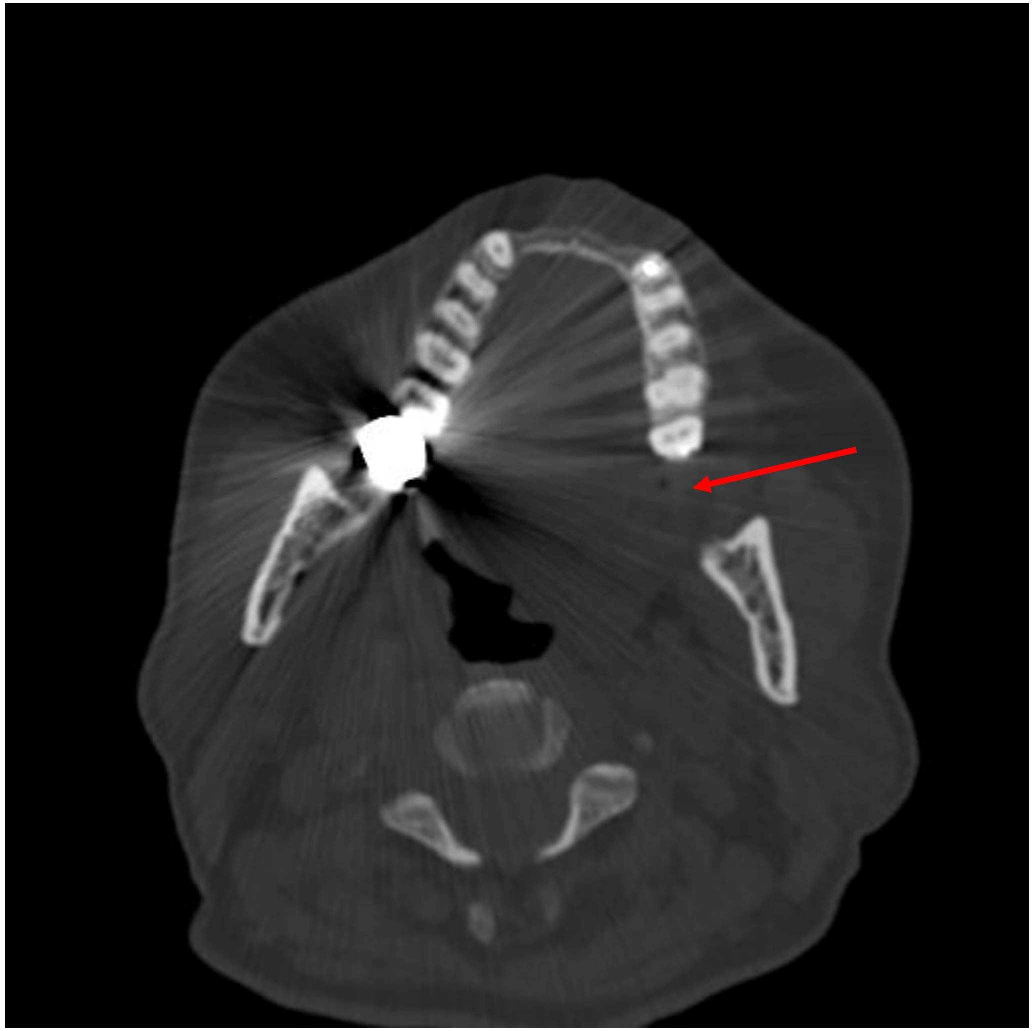

Path 2, edentulous (so no tooth # to be recorded)

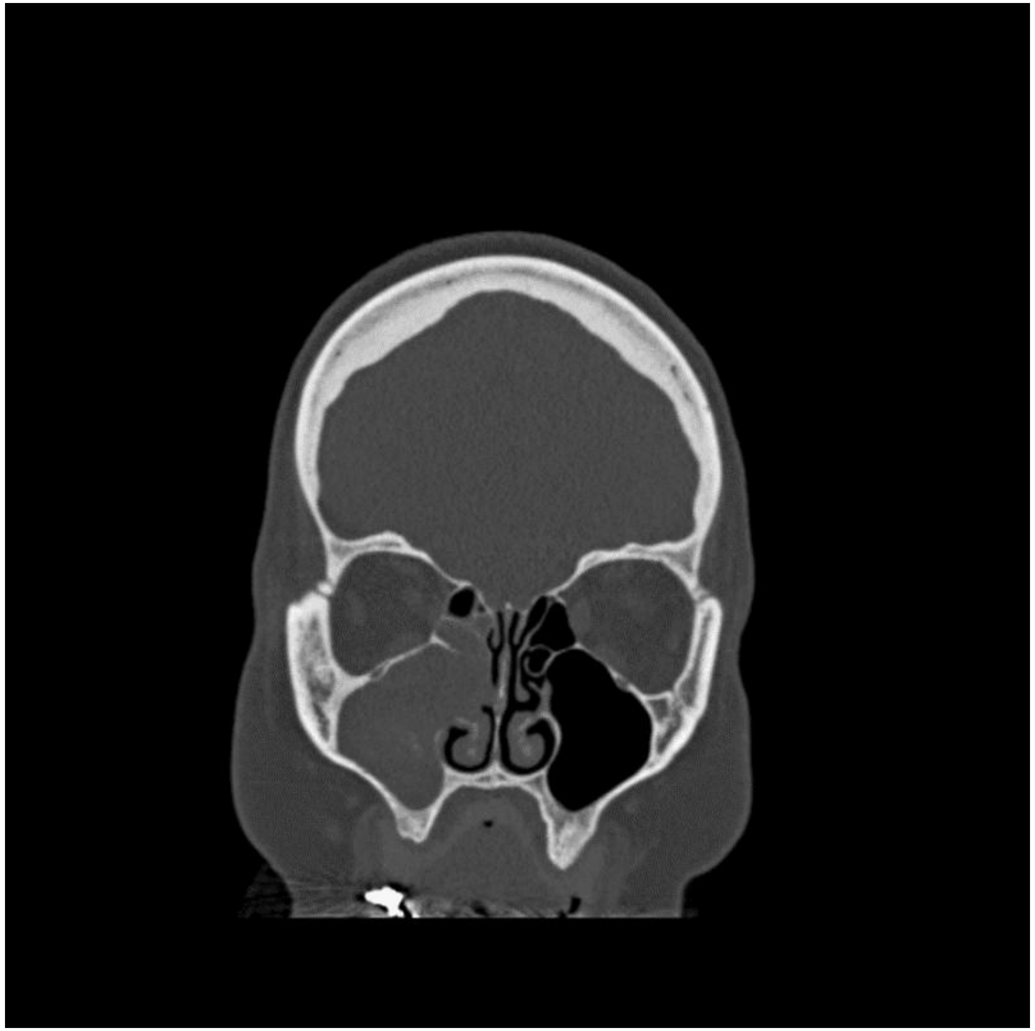

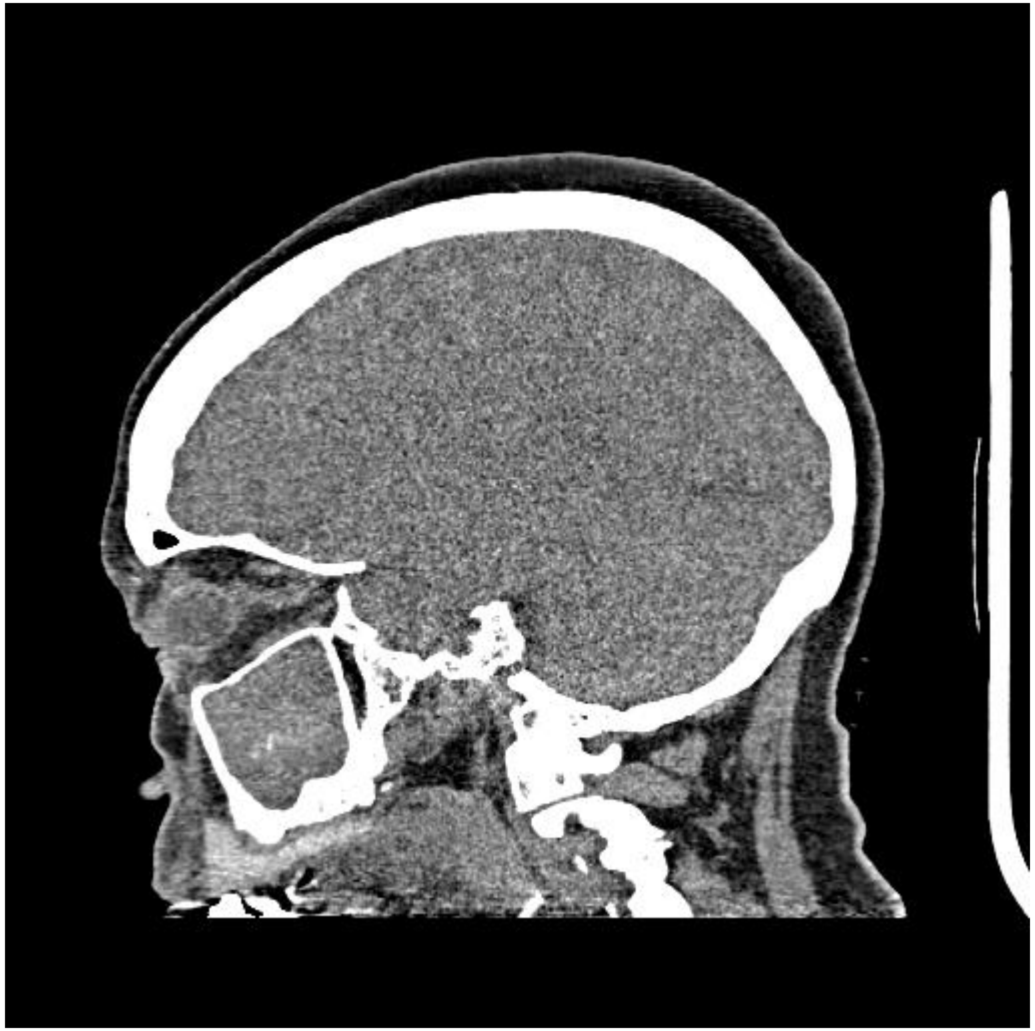

Path 3 (confirmed by Oral Exam!)

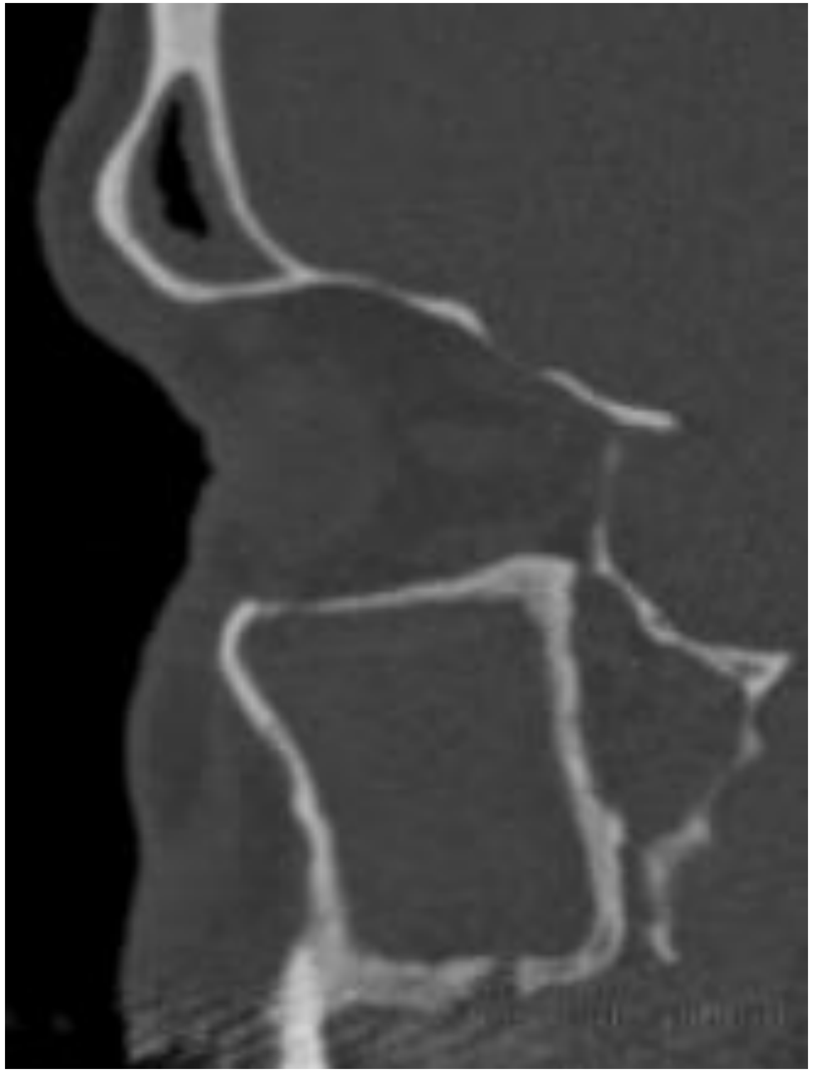

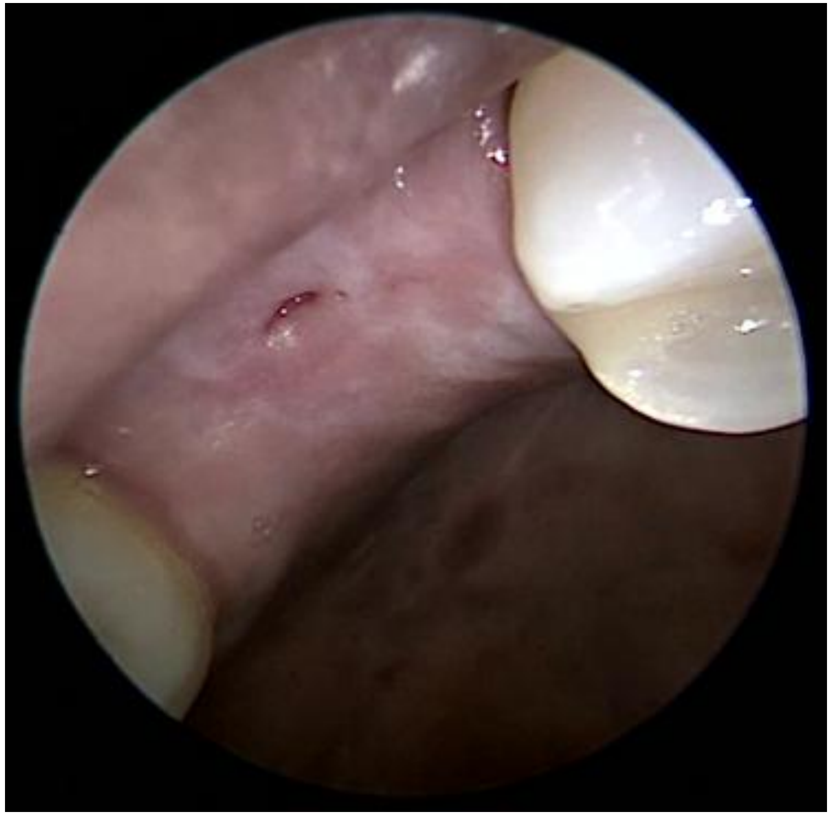

Path 4, Marginal periodontitis (NOT endodontic origin)

- For study, code this either if confirmed by dental specialist, or if no dental records, patients who have substantial periodontal bone erosion, and not just a periapical lesion with minor periodontal erosion; otherwise consider those with periapical and periodontal erosion to be apical periodontitis (more commonly the source)
- Ideally would have endodontic testing to rule out pulpal necrosis as the source of infection
- This will be a minor study limitation, but true marginal perio is an uncommon cause of ODS; also unlikely to confound this study's findings bc it is still an infectious source as opposed to indwelling dental metallic material

YES Perio

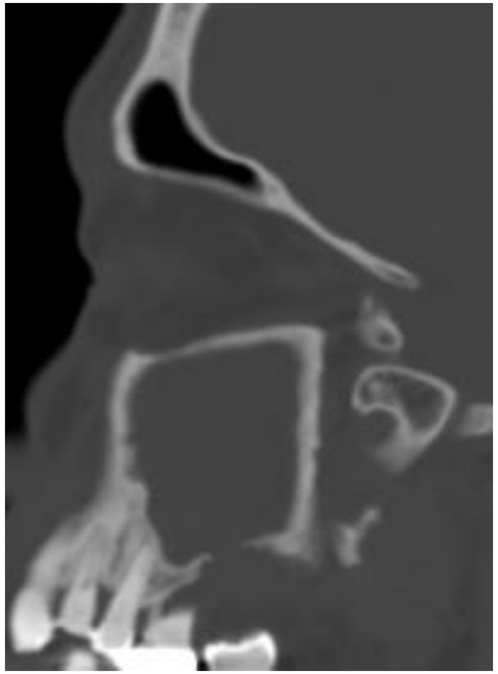

NOT perio

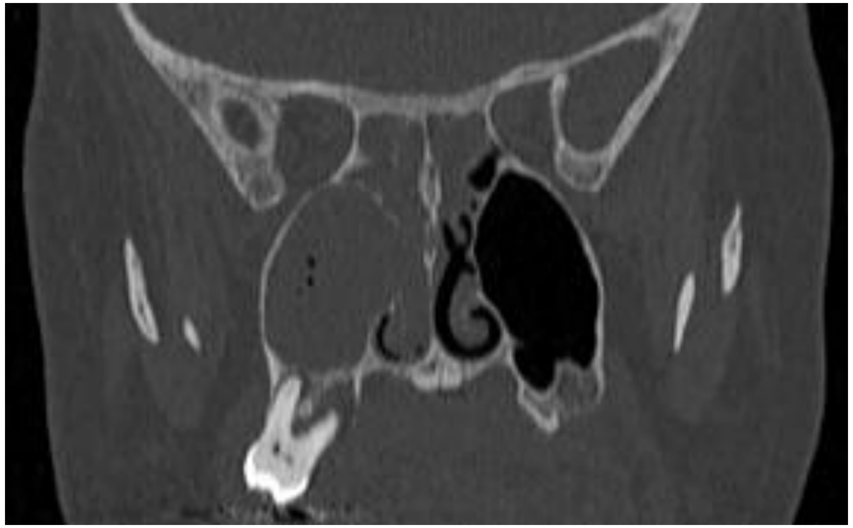

Paths 5 (left) and 6 (right); no need for tooth number(s) on these cases

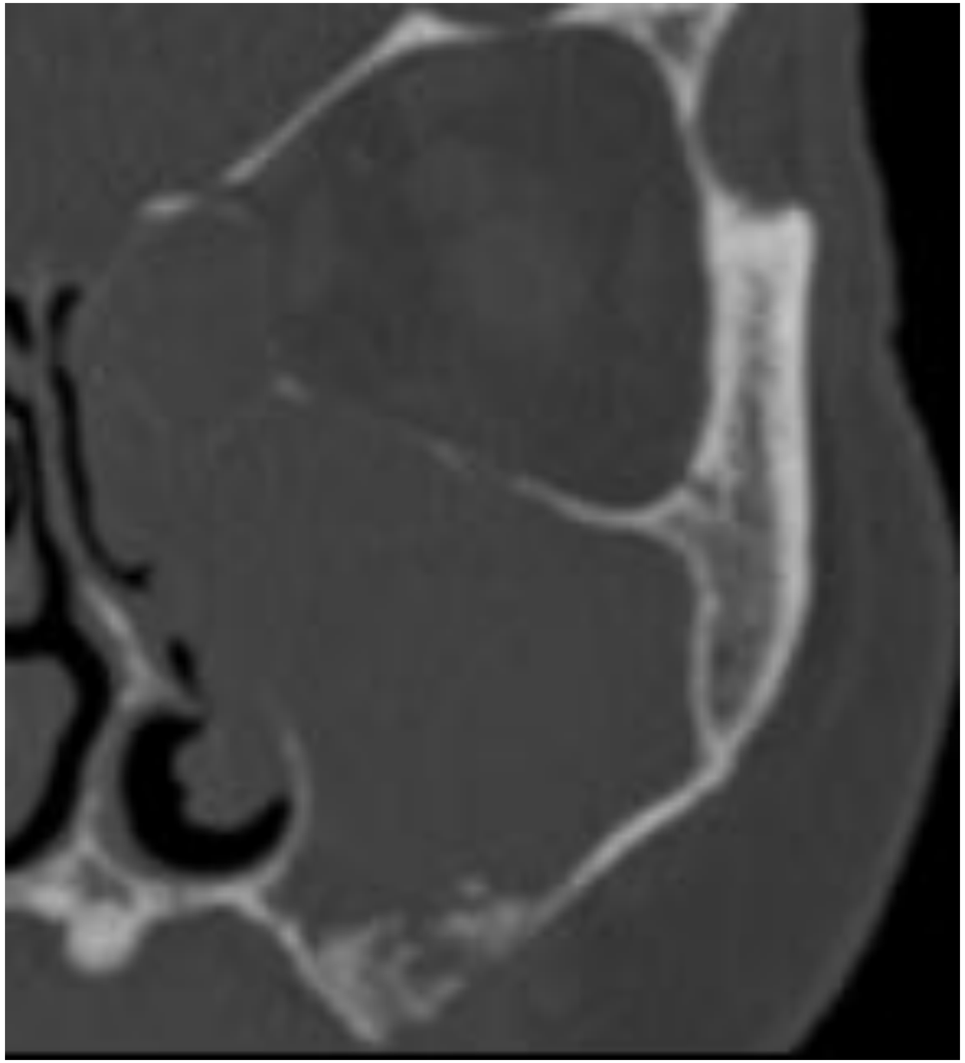

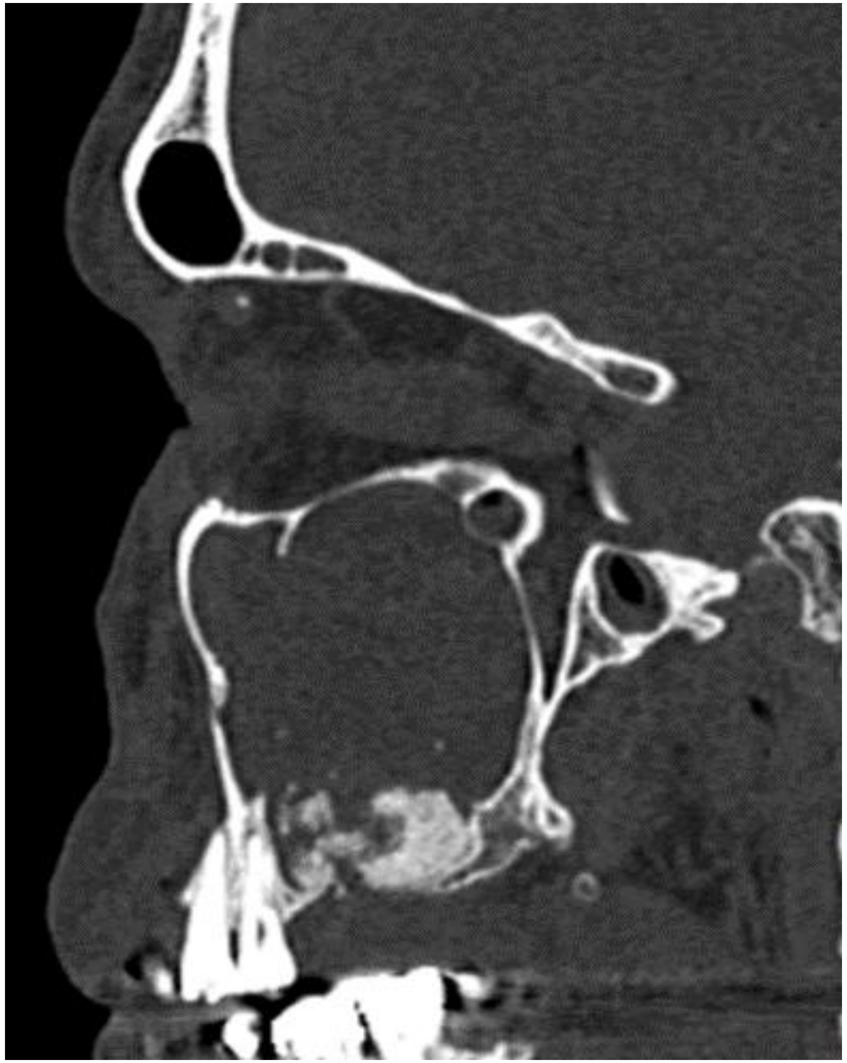

- If unsure whether extruded bone graft, check Hounsfield units (see slide at end of this PDF)
- NOTE: we still require fungal stains to include as fungal ball though

Dental path code 7; used axial + sagittal to prove that it was tooth #13

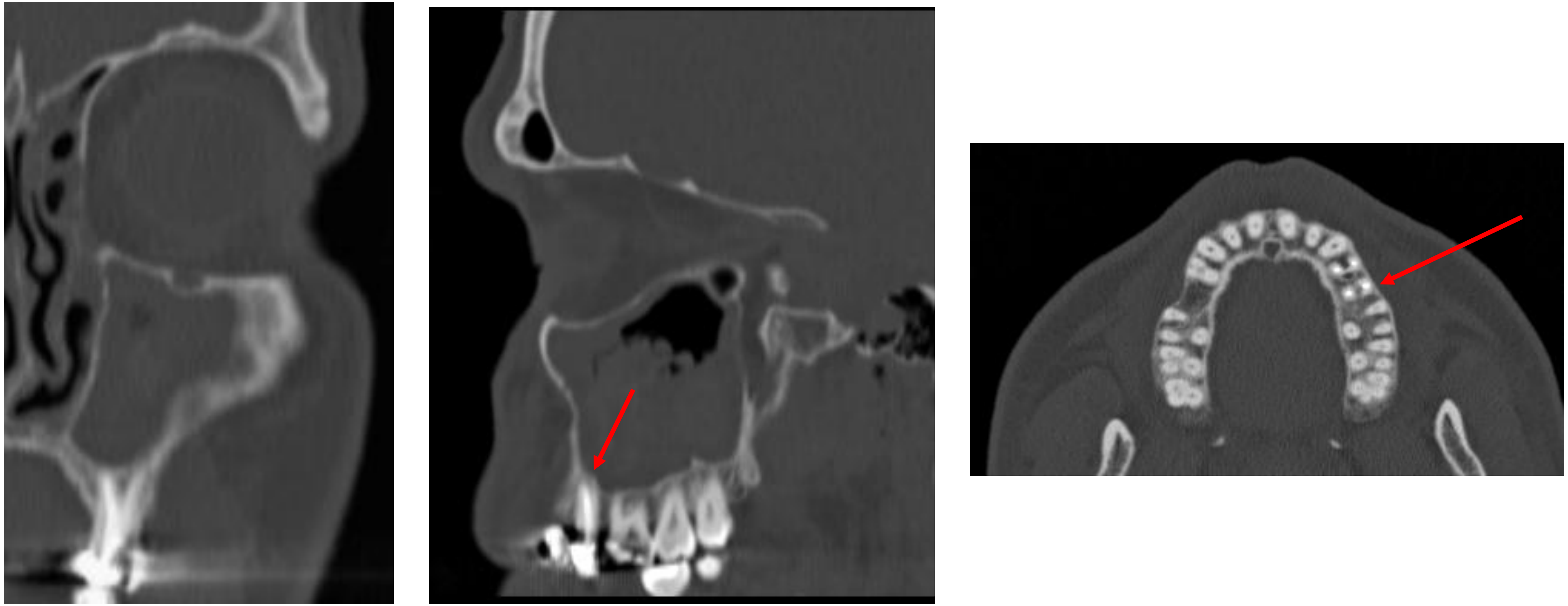

Path 8, tooth #2

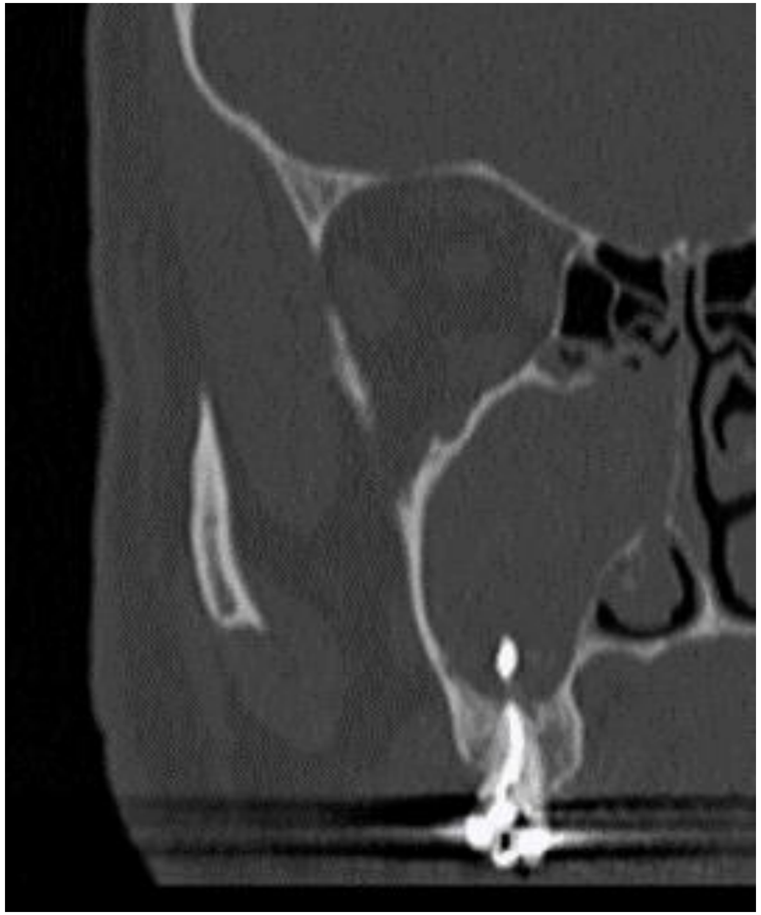
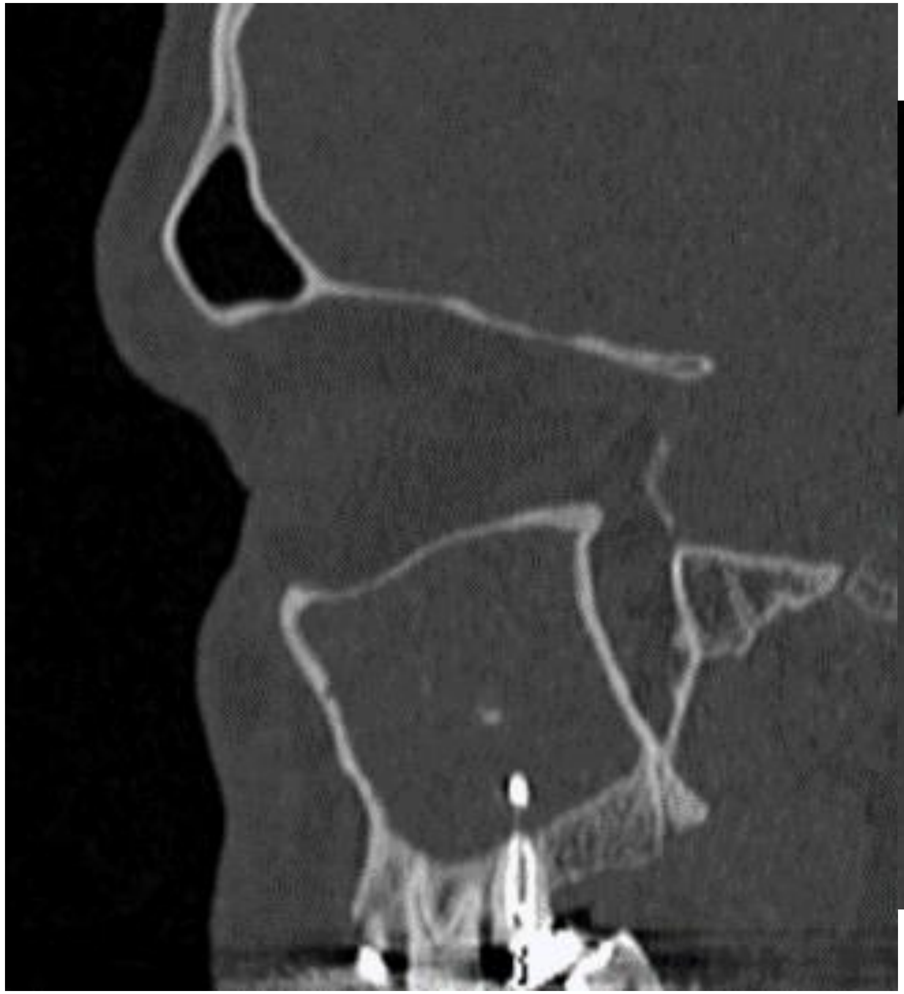
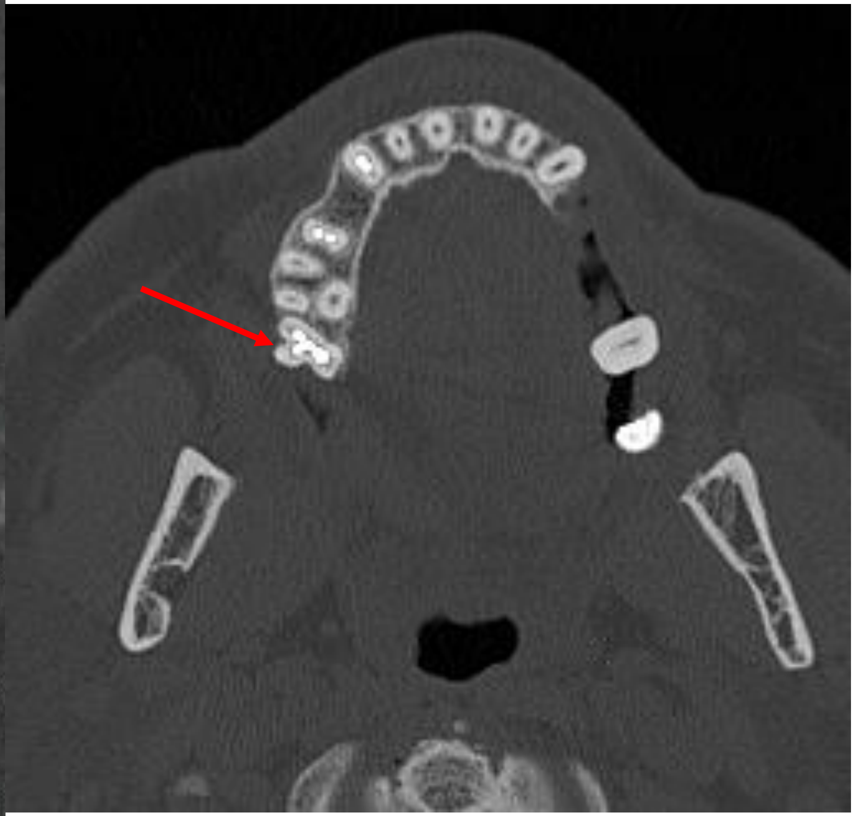

Path 9

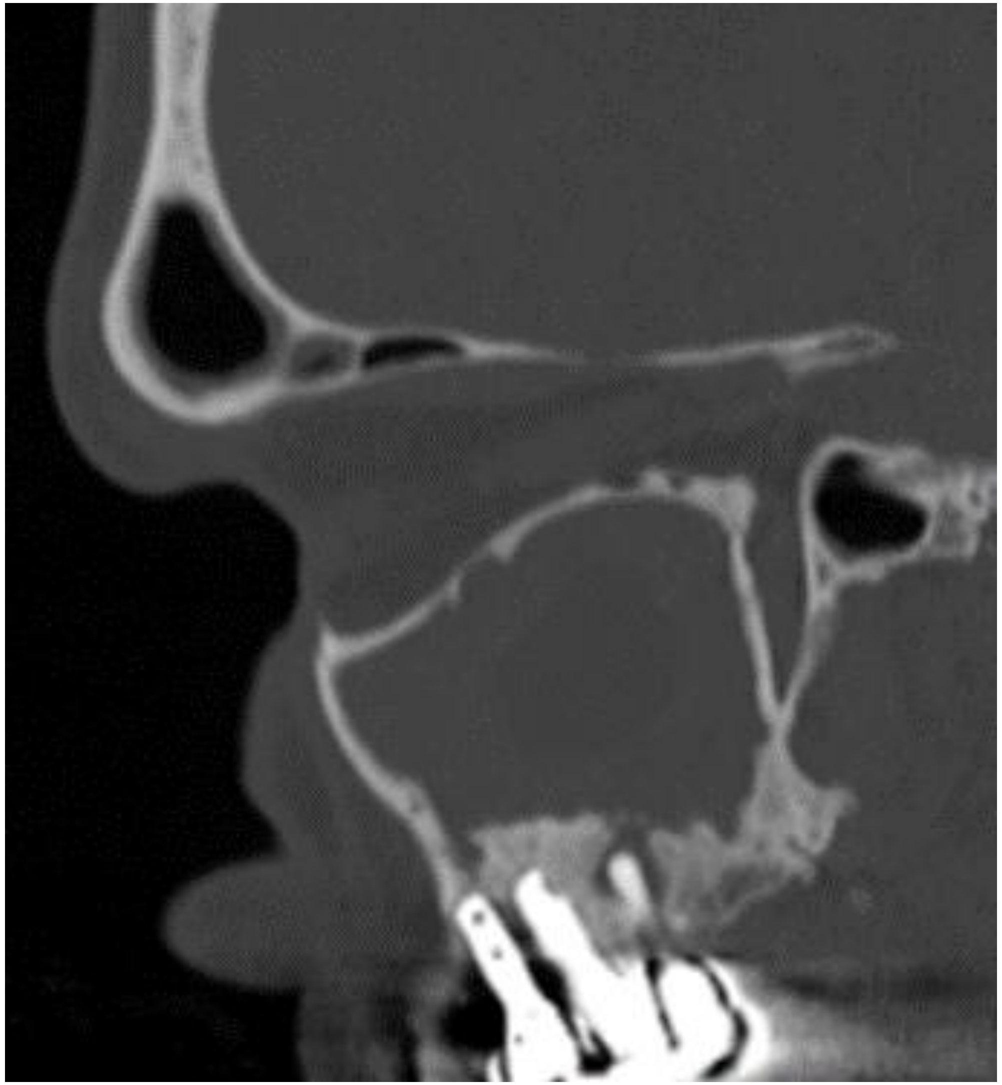

Coded as 10 for path, and had two at teeth #s 13 and 14

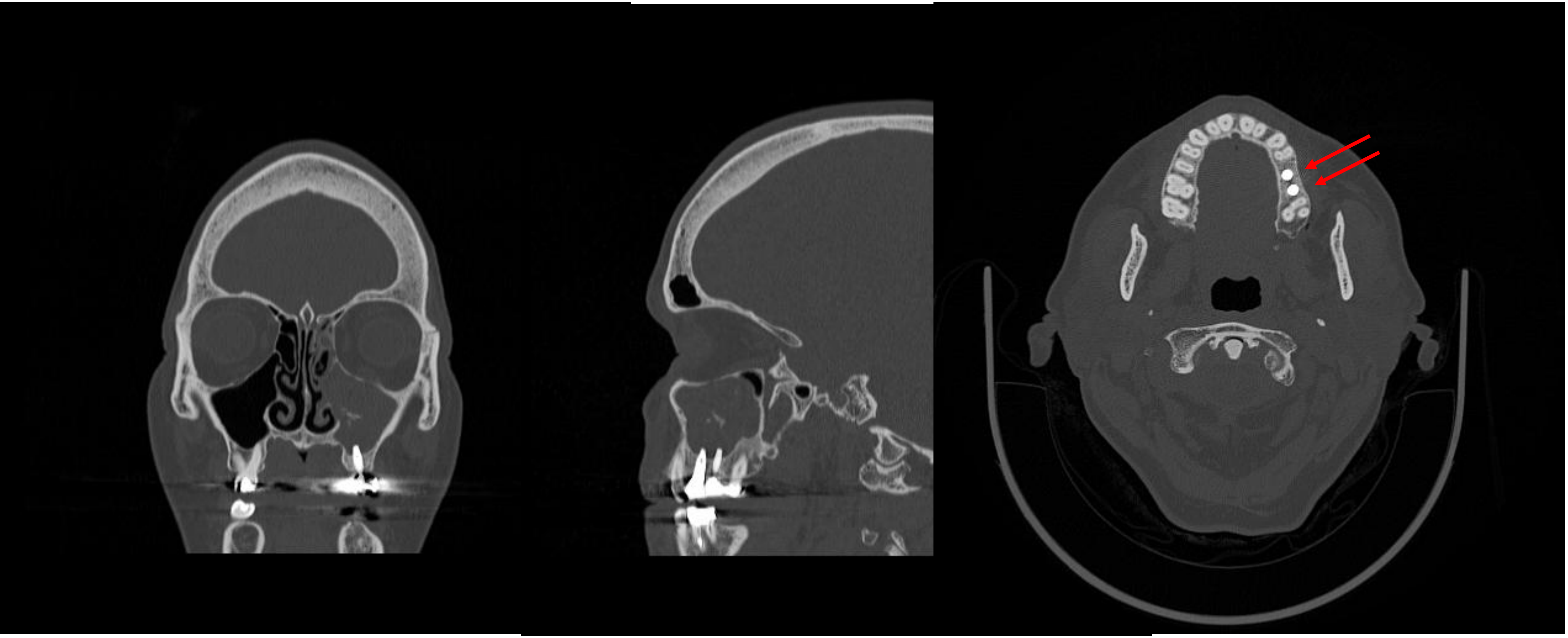

Path 11 (same as 12 in next image, except entirety of screw is within bone and not exposed to sinus mucosa or lumen)

Dental path code 12 both sides (plates or screw protruding into sinus lumen

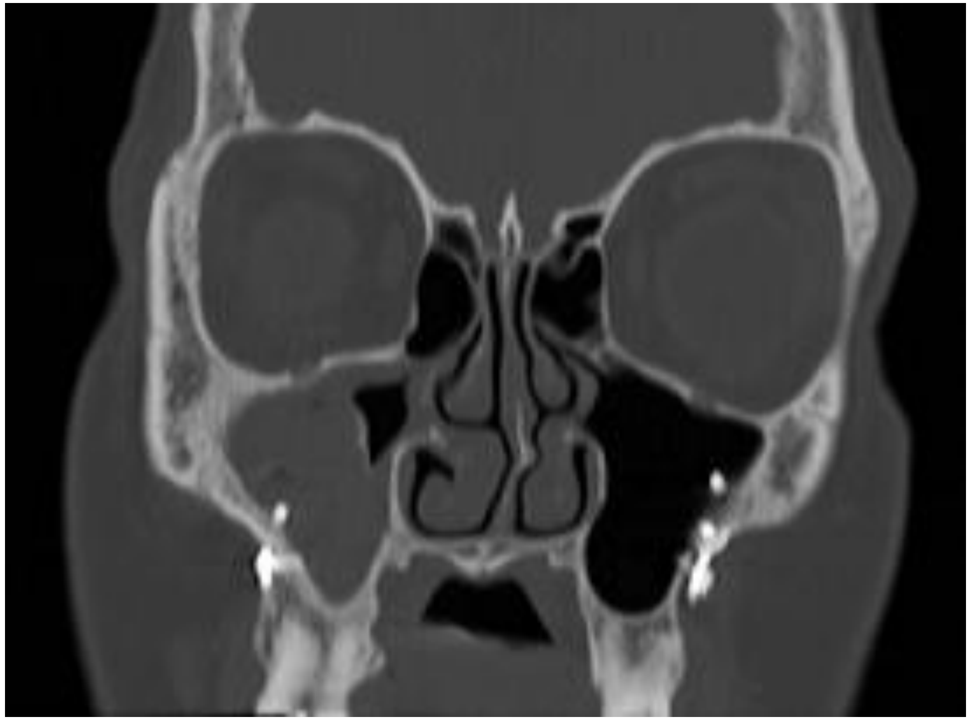

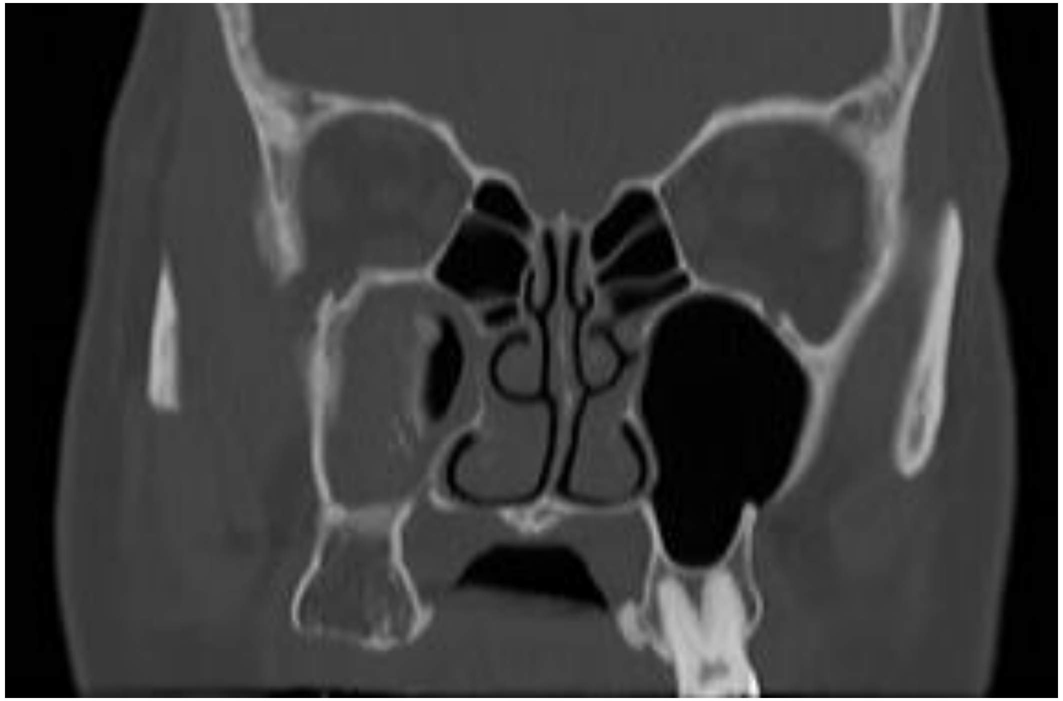

Determining whether dental materials are extruded into sinus on CT

- Use Hounsfield Units (HUs) on your PACS software, click on the material and make note of the HUs
  - Confirm dental material in the tooth or maxilla

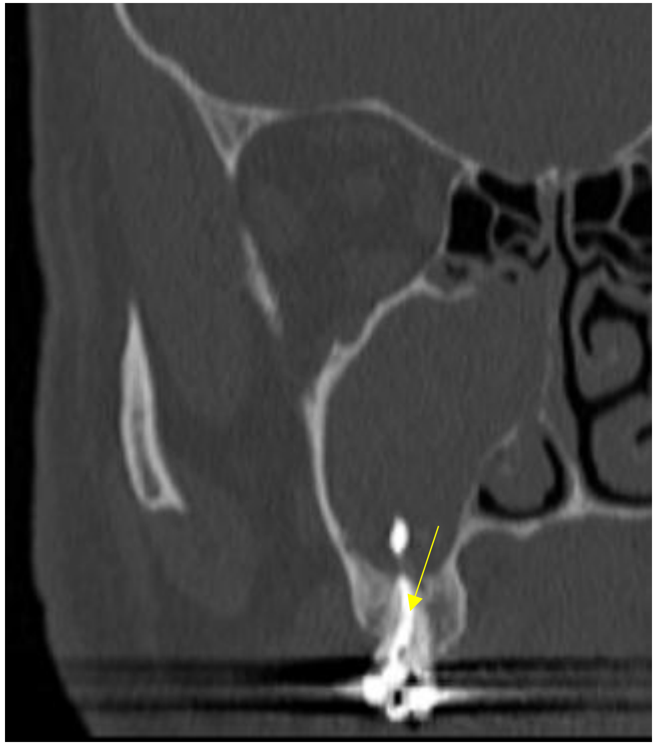

- Then check the diff materials in the lumen

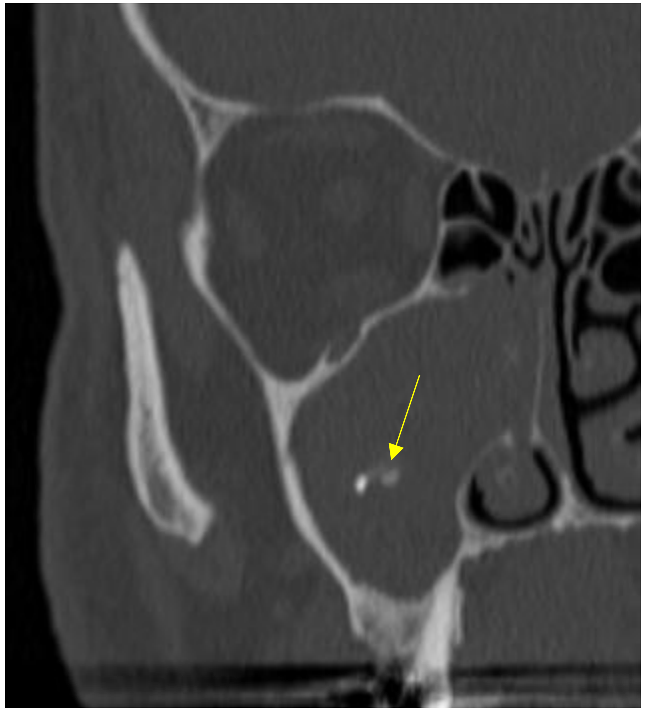

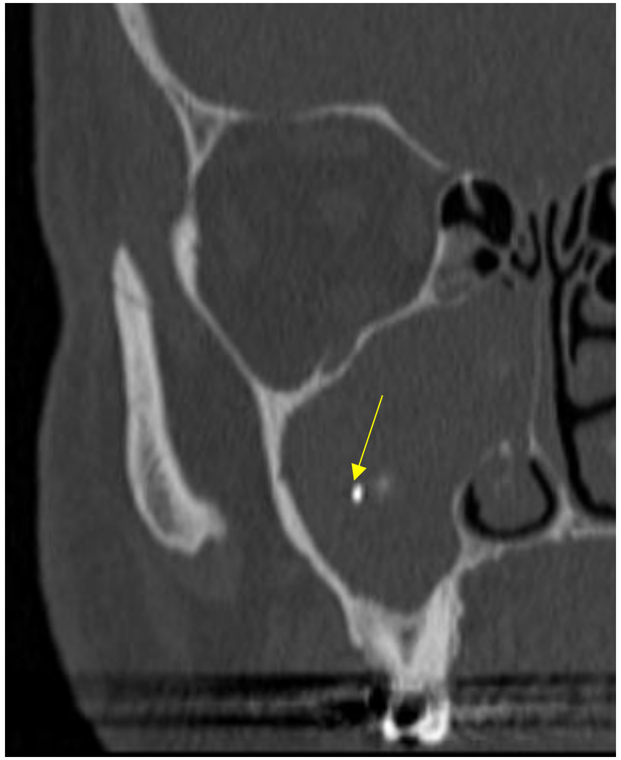

Supplement: Supplementary file 3 — Data S2: Examples of different dental pathologies and how to code them, plus how to determine whether intraluminal hyperdensities are likely from dental conditions using Hounsfield units. [file LARY-136-2913-s002.pdf]
