# Supplementary material for: Clinical Features and Dental Pathologies in Maxillary Sinus Fungal Balls and Odontogenic Sinusitis
Source: Laryngoscope. 2026 Feb 7;136(7):2913–24. doi: 10.1002/lary.70429 (PMC13253162; doi:10.1002/lary.70429)
Supplement: Supplementary file 4 — Table S1: Reasons for excluding certain patients from maxillary sinus fungal ball (MSFB) and odontogenic sinusitis (ODS) cohorts. [file LARY-136-2913-s005.docx]

**Supplemental Table I**

| **Excluded patients from MSFB group (n=36)** |
| --- |
| *Reasons for exclusion* |
| 14 - no staining to confirm fungal hyphae |
| 7 - bilateral MSFB |
| 7 - suspected MSFB on CT, but negative fungal stains |
| 4 - contralateral maxillary sinus opacification |
| 1 - inadequate viewing of maxillary dentition to determine dental pathology |
| 1 - invasive fungal sinusitis |
| 1 – maxillary sinus malignancy |
| 1 – CT imaging not available to review |
| **Excluded patients from ODS group (n=39)** |
| *Reasons for exclusion* |
| 24 - no staining to confirm or refute fungal hyphae |
| 13 - positive fungal stains |
| 2 - bilateral ODS |

Reasons for excluding certain patients from maxillary sinus fungal ball (MSFB) and odontogenic sinusitis (ODS) cohorts.
