# Supplementary material for: Clinical Features and Dental Pathologies in Maxillary Sinus Fungal Balls and Odontogenic Sinusitis
Source: Laryngoscope. 2026 Feb 7;136(7):2913–24. doi: 10.1002/lary.70429 (PMC13253162; doi:10.1002/lary.70429)
Supplement: Supplementary file 5 — Table S2: Comparisons of different dental pathologies and pathology groups between maxillary sinus fungal balls with odontogenic conditions (MSFBO) with versus without maxillary sinus (MS) purulence. MS, maxillary sinus; OAF, oroantral fistula; PAL, periapical lesion; RCT, root canal treatment. Bold p values bolded were statistically significant. [file LARY-136-2913-s003.docx]

| **Dental Pathologies** | **MSFBO with MS Purulence**  **(N = 74)** | **MSFBO without MS Purulence**  **(N = 67)** | **Adjusted P-value** |
| --- | --- | --- | --- |
| **No dental pathology** | 0 (0.0%) | 0 (0.0%) | - |
| **Infectious or possible infectious dental pathologies**  **[n (%)]** | 42 (56.8%) | 47 (70.1%) | 0.141 |
| Apical periodontitis with PAL | 16 (21.6%) | 12 (17.9%) | 0.734 |
| Post-extraction without OAF | 21 (28.4%) | 34 (50.7%) | **0.011** |
| Post-extraction with OAF | 2 (2.7%) | 0 (0.0%) | 0.521 |
| Marginal periodontitis | 3 (4.1%) | 3 (4.5%) | 1.000 |
| Bone graft without implant,  without graft  particle extrusion | 0 (0.0%) | 0 (0.0%) | - |
| Bone graft without implant, with  graft particle extrusion | 0 (0.0%) | 0 (0.0%) | - |
| **Indwelling dental metallic materials**  **[n (%)]** | 35 (47.3%) | 24 (35.8%) | 0.227 |
| Prior RCT ± PAL, without filling  material extruding into MS | 14 (18.9%) | 9 (13.4%) | 0.514 |
| Prior RCT ± PAL, with filling  material extruding into MS | 3 (4.1%) | 6 (9.0%) | 0.399 |
| Dental implant ± bone graft,  without protrusion into MS | 6 (8.1%) | 3 (4.5%) | 0.592 |
| Dental implant ± bone graft, with  protrusion into MS | 6 (8.1%) | 2 (3.0%) | 0.343 |
| Midface screws without protrusion  Into MS | 1 (1.4%) | 0 (0.0%) | 1.000 |
| Midface screws with protrusion  into MS | 4 (5.4%) | 4 (6.0%) | 1.000 |
| Free-floating metallic dental  foreign body in MS | 1 (1.4%) | 2 (3.0%) | 0.931 |

**Supplemental Table II:** Comparisons of different dental pathologies and pathology groups between maxillary sinus fungal balls with odontogenic conditions (MSFBO) with versus without maxillary sinus (MS) purulence. PAL, periapical lesion; OAF, oroantral fistula; RCT, root canal treatment; MS, maxillary sinus. Bold p-values bolded were statistically significant.
